# Redesigning a Web-Based Stakeholder Consensus Meeting About Core Outcomes for Clinical Trials: Formative Feedback Study

**DOI:** 10.2196/28878

**Published:** 2021-08-19

**Authors:** Roulla Katiri, Deborah A Hall, Derek J Hoare, Kathryn Fackrell, Adele Horobin, Nóra Buggy, Nicholas Hogan, Pádraig T Kitterick, Core Rehabilitation Outcome Set For Single-Sided Deafness (CROSSSD) Initiative

**Affiliations:** 1 Hearing Sciences Mental Health and Clinical Neurosciences School of Medicine, University of Nottingham Nottingham United Kingdom; 2 National Institute for Health Research (NIHR) Nottingham Biomedical Research Centre (BRC) Ropewalk House Nottingham United Kingdom; 3 Audiology Department Mater Misericordiae University Hospital Dublin Ireland; 4 Department of Psychology School of Social Sciences Heriot-Watt University Malaysia Putrajaya Malaysia; 5 Nottingham University Hospitals NHS Trust Queen’s Medical Centre Nottingham United Kingdom; 6 Wessex Institute University of Southampton University Road Southampton United Kingdom

**Keywords:** COVID-19, nominal group technique, formative research, patient participation, persons with hearing impairments, mobile phone

## Abstract

**Background:**

Clinical trials that assess the benefits and harms of an intervention do so by measuring and reporting *outcomes*. Inconsistent selection and diversity in the choice of outcomes make it challenging to directly compare interventions. To achieve an agreed core set of outcomes, a consensus methodology is recommended, comprising a web-based Delphi survey and a face-to-face consensus meeting. However, UK government regulations to control the pandemic prohibited plans for a face-to-face consensus meeting as part of the Core Rehabilitation Outcome Set for Single-Sided Deafness (CROSSSD) study.

**Objective:**

This study aims to evaluate the modifications made by the CROSSSD study team to achieve consensus using web-based methods, but with minimal deviation from the original study protocol.

**Methods:**

The study team worked with health care users and professionals to translate the planned face-to-face consensus meeting in a web-based format, preserving the key elements of the nominal group technique. A follow-up survey gathered evaluation feedback on the experiences of the 22 participating members. Feedback covered premeeting preparation, the process of facilitated discussions and voting, ability to contribute, and perceived fairness of the outcome.

**Results:**

Overall, 98% (53/54) of feedback responses agreed or strongly agreed with the statements given, indicating that the web-based meeting achieved its original goals of open discussion, debate, and voting to agree with a core outcome set for single-sided deafness. Hearing-impaired participants were fully engaged, but there were some methodological challenges. For the participants, challenges included building rapport, understanding, and delivering the tasks in hand. For the study team, challenges included the need for thorough preparation and management of the unpredictability of tasks on the day.

**Conclusions:**

Sharing our experiences and lessons learned can benefit future core outcome set developers. Overcoming the challenges of delivering a web-based consensus exercise in the face of the pandemic can be applied more generally to maximize inclusiveness, enhance geographical access, and reduce research costs.

## Introduction

### Background

When choosing a treatment for a disease or disorder, health care users, health care professionals, and other stakeholders need evidence of the benefits and harms of the treatments. Clinical trialists gather evidence by comparing and contrasting the benefits and harms (outcomes) of medical, surgical, or behavioral interventions. However, clinical trials evaluating interventions often measure and report different outcomes [1], making it challenging to synthesize evidence to inform recommendations on clinical management.

To address the inconsistency of outcome selection, clinical trialists recommend developing a *core outcome set* (COS). A COS prescribes the minimum set of outcomes that should be measured and reported when testing an intervention for a given health condition. The Core Outcome Measures in Effectiveness Trials (COMET) initiative has published a handbook to promote good practice in COS development methods [2]. The conventional process involves structured communication with patients and clinicians using a Delphi survey administered as a questionnaire [3], followed by a smaller scale consensus meeting [2]. Although the questionnaire can be administered on the internet, the consensus meeting is typically face-to-face [2,4], consistent with other applications of the nominal group technique (NGT) in the context of health care research [5].

### Objectives

In line with the standard process, our project team (Core Rehabilitation Outcome Set for Single-Sided Deafness [CROSSSD]) had planned a web-based Delphi survey followed by a face-to-face consensus meeting [6]. However, we had to revise these meeting plans to comply with the travel and physical distancing restrictions imposed by the UK government in 2020 in response to the COVID-19 pandemic. There is limited information about web-based qualitative data gathering from groups in health care [7-10], and its evaluation from the participant perspective appears to be somewhat minimal [11]. Given that CROSSSD is about people with single-sided deafness (SSD), the web-based methods adopted had to be accessible to people with possible communication difficulties and suitable for data gathering, adding an extra layer of considerations.

The primary aims of this study are (1) to describe how we redesigned the consensus meeting format from face-to-face to web-based and (2) to evaluate stakeholder experiences.

## Methods

### Participants

A face-to-face consensus meeting was organized as per protocol [6] to take place in London, United Kingdom, on March 19, 2020. A total of 22 participants were invited (6/22, 27% health care users; 8/22, 36% health care professionals; 2/22, 9% public research partners, who had first-hand or lived experience of SSD; 1/22, 5% patient involvement manager; 2/22, 9% facilitators; 2/22, 9% members of the study management team; and 1/22, 5% observer). Overall, 68% (15/22) of participants traveled from within the United Kingdom and 32% (7/22) participants from Europe. On cancelation of the face-to-face meeting, we invited participants to continue their involvement. Methodological changes required only notification of a nonsubstantial amendment to the Nottingham 2 Research Ethics Committee, who approved the study. Examples of these changes included (1) amendment of the participant information leaflet to say *web-based consensus meeting*, (2) recording individual consent on the internet, and (3) extending the study end date.

Of the original group of participants, 1 health care user could not attend the rescheduled date (July 7, 2020) and 2 health care professionals did not respond to the invitation to the web-based meeting. A replacement health care user was recruited to maintain the balance across stakeholder groups. One additional facilitator was also recruited so that the web-based discussion groups were manageable.

A commercial representative based in Denmark and a US-based clinical researcher asked the CROSSSD team if they could join the meeting and so were invited to attend as nonparticipating observers. Therefore, the revised group of participants comprised 23 individuals, of which 12 (52%) were eligible to vote because they had completed both rounds of the Delphi survey. Participants consented to participate in the consensus meeting by completing a web-based form. Voting during the consensus meeting was conducted using hyperlinks to Jisc web-based surveys [12].

### Redesigned Meeting

We used Microsoft Office Teams [13] for web-based discussions because (1) it was supported by the study sponsor; (2) it was freely available; and (3) it had desirable features, including a gallery view of all participants, a chat function, live caption ability, and audio recording. Optional one-to-one practical software tutorials were offered to all participants before the meeting to ensure that all necessary functionality was accessible and understood by participants. A discretionary virtual coffee morning was held the week before the meeting to enable participants to test the technology and meet each other socially. A total of 22 participants attended (1/22, 5% chairperson; 6/22, 27% health care users; 6/22, 27% health care professionals; 2/22, 9% public research partners; 1/22, 5% patient involvement manager; 2/22, 9% facilitators; 2/22, 9% other members of the study team; and 2/22, 9% observers). Overall, 3 health care professionals, 2 observers, 1 facilitator, and 1 study team member could not attend because of work commitments.

The public research partners, patient involvement manager, and facilitators with experience in conducting face-to-face COS consensus meetings and qualitative research contributed to the planning of the web-based meeting, including its structure, timing, preparatory activities, communication strategies, discussion points, and voting techniques. Public research partners helped to enhance accessibility for those with hearing difficulties, drawing upon their own lived experience, as per recommendations when designing COS studies [2,14]. These enhancements included meeting etiquette (eg, use the *raise hand* function and wait your turn), chairing (eg, making the facilitators aware of their role, ways to resolve conflict, and adhering to the agenda), accessibility (eg, enabling the automatic captions), and troubleshooting (eg, use the chat function or exit and re-enter the software).

In line with the approach advocated by COMET, and to obtain qualitative information from our participant group in a structured manner [15], an NGT approach [16] was adopted. NGT allows groups to explore and thoroughly discuss issues in hand, identify, rank, and rate various problem dimensions with limited researcher influence or interference [5,7]. Conventionally, NGT comprises the following steps: (1) a chairperson introduces the group, sets ground rules, and explains the purpose of the meeting and procedures for the day; (2) the chairperson states the question and encourages each participant to individually reflect and brainstorm; (3) with the help of a facilitator, participants have an opportunity to discuss and clarify ideas; and (4) participants evaluate the ideas and vote anonymously for the *best ideas*. In CROSSSD, steps (3) and (4) were conducted in three parallel subgroups. Each facilitator presented the main discussion points using predetermined guidance (Multimedia Appendix 1) before voting. When consensus was required, an additional step (5) shared the voting results with the group and provided the opportunity to discuss and vote again. In this study, the results were presented using histograms embedded in PowerPoint slides.

CROSSSD extended the NGT by requesting participants to engage in certain activities in advance, namely, (1) inviting them to meet the group at the discretionary coffee morning; (2) introducing the meeting purpose, procedures, and Delphi survey results via an information pack (Multimedia Appendix 2), PowerPoint slides (Multimedia Appendix 3), and a prerecorded presentation (Multimedia Appendix 4); and (3) asking them to vote for three outcomes they considered crucial to include in the COS before the day of the consensus meeting. Further modifications were (4) introducing a structured ice-breaker activity and (5) providing subgroup support from a public research partner or patient involvement manager and facilitator. The subgroup composition was predetermined to achieve a balance of stakeholder perspectives and to facilitate the efficient organization of subgroup discussions on the day of the meeting.

**Figure 1:**
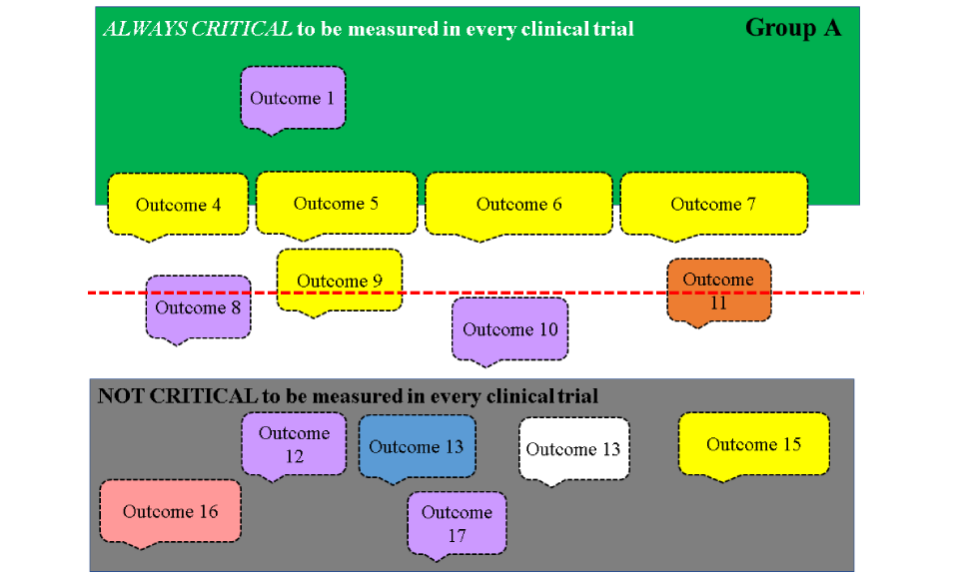
The PowerPoint slide used to provide a visual display of the outcomes for consideration and to assist the facilitators when guiding discussions or summarizing subgroup discussions.

During the consensus meeting, which was 7 hours long with three 30-minute-long breaks, a series of discussion and voting steps reduced the pool of candidate outcomes to a final COS. The first step was to present the results from the *top three outcomes* survey conducted before the meeting and asked whether participants agreed to exclude those outcomes that had not been selected by anyone to be in their top three. The voting options were *agree*, *disagree*, or *unsure*. Next, participants were asked to consider the remaining outcomes and to identify five outcomes that they considered critical to be measured in every clinical trial of interventions for SSD. During subgroup discussions, the facilitators moved the outcomes around a shared visual display to reflect discussions (Figure 1). The green zone included outcomes considered *always critical*, the gray zone included outcomes considered *not critical*, and the intermediate zone was for those with mixed opinions or not yet discussed.

When participants returned to the full group, they were then asked to vote whether they would exclude those outcomes considered *not critical* (ie, in the gray zone). This process was repeated again after the whole group and subgroup discussions to reduce the list of candidate outcomes. Finally, participants considered the remaining outcomes that had not yet been voted *in* or *out* and voted on whether the *always critical* set should form the COS for SSD interventions. Applying the criterion of 70% agreement as per protocol [6], at least nine of the 12 participants agreed for any decision to be carried out.

For formative feedback, all 12 voting participants were asked to complete a web-based consensus meeting evaluation (Multimedia Appendix 5), adapted from the COMET Initiative [17]. Participants responded to six statements on the premeeting information, their experience of the consensus meeting, and fairness of the outcome using a 5-point Likert scale (strongly agree, agree, neither, disagree, or strongly disagree) and open text boxes for further comments. Each statement is described in the *Results* section. Two further open text boxes sought feedback on the practical arrangements for the meeting and suggestions for improvement. All other meeting participants were invited to respond to a modified version of the evaluation involving the open text comments only.

## Results

### Overview

Formative feedback was received from 75% (9/12) of voting participants (4 health care users and 5 health care professionals) and 40% (4/10) of the study team (2 public research partners, 1 patient involvement manager, and 1 facilitator). To illustrate the key points, many of the comments from one health care user (HU2) who was highly articulate are shared below. These views were confirmed by the study team’s reflections.

### Examples of Formative Feedback

Concerning whether the information provided in advance was helpful, all voting participants (9/9, 100%) agreed or strongly agreed. One said as follows:

Communication by the organisers with participants in advance of the meeting was absolutely first class with ample opportunity offered for consultation about any areas of concern and clarification when needed was always offered promptly and with considerable patience.

HU2

The facilitator commented as follows:

The pre-meeting information was very thorough. The Teams meeting was extremely valuable—contrary to my expectations. I expected this to be a confirmatory meeting; instead the facilitators highlighted aspects of the schedule which might not work so well, and everyone made contributive comments on how to make the online work. As a result some fundamental changes were made but we all felt we input into this process.

The patient involvement manager agreed: “I had time at a prep-meeting to ask questions and to clarify the procedures for the day.” Regarding whether the process used to agree with the COS was satisfactory, most participants (8/9, 89%) agreed or strongly agreed:

The process was particularly rigorous. The highest level of support was available from the leaders of the meeting but there was no heavy-handed intervention.

HU2

One public research partner highlighted the benefit of preparation:

The meeting had to be reconfigured to proceed remotely and this was handled exceptionally well [...] a lot of thought went into it and it showed, [...] technical support was provided promptly and without fuss or exasperation.

Only one health care professional indicated that the process could have been improved by reorganizing the subgroups during the day:

I think the discussion in each group was influenced by the members, so some mixing would have helped [...] in the end there was a reasonably good outcome though.

All participants (9/9, 100%) agreed or strongly agreed that meeting facilitation was satisfactory. Comments included were as follows:

The leaders were superb facilitators and every participant was made to feel as if their voice was important.

HU2

The facilitators were absolutely first class professionals and I felt privileged to have had the opportunity of working with them.

HU2

Again, all participants (9/9, 100%) agreed or strongly agreed that they felt able to contribute to the meeting. One supporting comment was as follows:

Everyone without exception was encouraged to participate fully at the meeting and the facilitators displayed great sensitivity to the needs of each individual contributor. From a personal point of view, I was concerned that the technology used for the meeting might impede successful and effective communication, but it didn’t, thanks to the watchful eye of the leaders of the meeting who actively encouraged free expression from every participant while at the same time subtly guiding the proceedings to ensure maintenance of a structure which would lead to fulfilment of the consensus meeting’s objectives. I would also like to add that a very fine rapport between participants was quickly established.

HU2

A facilitator indicated that past experience was important:

It had helped having been involved previously in facilitating three face-to-face COS consensus meetings. I drew heavily from that previous experience.

Similarly, all participants (9/9, 100%) agreed or strongly agreed that they felt comfortable communicating their views. For example:

People taking part demonstrated great empathy for their fellows and there was a heart-warming sense of co-operation [...] delegates had ample opportunity to share their ‘story’ [...] I was made to feel like a person of value with something significant to contribute and I was particularly struck by the very high level of respect which people demonstrated for each other.

HU2

Finally, all participants (9/9, 100%) agreed or strongly agreed that the consensus meeting produced a fair result. One said:

There was at times quite heated debate, but I believe that a consensus was finally reached which reflected the majority view.

HU2

Subsequently, a Jisc survey of the wider stakeholder community confirmed 97% (89/92) agreement with the COS decisions made during the consensus meeting.

### Participant Preferences

One of the major recurring themes was the preference for social interactions over web-based meetings. Two health care professionals said as follows:

Given the circumstances, this was a perfect solution, nevertheless I missed the social interactions.

I personally don’t like remote meetings. I feel they stifle free speech and the normal interactions and debate cannot happen in the same way.

Nothing could have been better other than the face-to-face interaction [...] however, we enjoyed the benefits of the next best thing and there were also clearly some advantages in having a virtual workshop.

HU2

Another lesson concerned time management. At one point in the afternoon, there was some misunderstanding about the length of a break and when to reconvene, and this lost about 10-15 minutes of the schedule. Clear communication can avoid such issues. More generally, different stakeholders concurred that there was too little time for discussion. One health care professional said as follows:

I felt more time for each group to discuss the reasons behind their selected outcomes with the other groups, and to explain why they have selected one above another would have been useful...I enjoyed the in-group discussion, but felt the between-group discussions were a bit rushed/short.

The patient involvement manager commented as follows:

We were a little rushed; not enough time for whole group discussions and voting.

With regard to improvements to the web-based meeting, one public research partner recommended to plan more time at the end for discussion of the COS:

I felt that maybe a safety net or reserve of one hour might have been added to the end.

Taking fatigue into account, the patient involvement manager suggested a debriefing might be deferred to a later date and be organized in the same way as the discretionary coffee morning to “allow participants to reflect with each other and to feel an appropriate ‘closure’, rather than a very intense day followed by a very quick ‘goodbye’.”

## Discussion

### Principal Findings

To the best of our knowledge, no previous COS development studies have adopted a fully web-based consensus methodology. On the basis of participant feedback, the premeeting preparation, choice of software, and approach to facilitation on the day proved effective in ensuring meaningful participant engagement. On balance, we conclude that the web-based method adopted for this meeting was successful and produced a result that was genuinely reflective of the group consensus.

We aim to create a safe environment with a *sense of belonging* to help participants feel valued, so they might share information more spontaneously. Although SSD is known to cause difficulty in following conversations in group situations, which can lead to listening fatigue and withdrawal [18], none of the participants mentioned such disadvantages. We suggest that the web-based meeting overcame these issues, as participants could access software features, including live subtitling on demand and *raise your hand*, which gives a clear right to conversation turn-taking that is not always achievable in face-to-face meetings [19]. Therefore, the structured discussions built into the web-based schedule were considered an advantage for this participant group.

### Lessons Learned

Web-based meetings, unlike face-to-face meetings, can confer some advantages for participants to contribute effectively [8]. Three general methodological approaches have the greatest positive impact. The first approach concerned meeting planning and preparation. We followed recommendations to seek input from public research partners at all stages of COS development, drawing in perspectives based on the lived experience of SSD [2,14]. Their suggestions included lengthening the subgroup discussion time, screen sharing of the visual display, and sharing each subgroup’s slide (Figure 1) with the meeting chair. Experienced facilitators pre-empted potential challenges. For example, a suggestion to add an activity prioritizing the outcomes within the COS was rejected after consultation with the facilitators because it was considered overambitious for the time scales. However, a suggestion to hold brief study team catch-ups during the breaks was endorsed, aiming to address any arising incidents and enhance participant contribution [8]. Detailed premeeting documentation informed participants about the process [7], setting clear expectations [20], and explaining the minimum participant requirements [11]. Participant feedback indicated that they appreciated this careful preparation. The second approach involved software training. Although all CROSSSD study facilitators and some participants had good prior working knowledge of the audiovisual technology chosen to closely mirror a face-to-face environment, some participants had no previous experience. To optimize interactions, as recommended by Flynn et al [10], we ensured that all participants joined with a camera, either via a computer, smartphone, or tablet. Guidance notes, one-to-one tutorials, and the virtual coffee morning offered an opportunity to learn and practice using the technology. Finally, the third approach involved the study team taking a number of steps to ensure satisfaction with the meeting arrangements. Numerous authors have recommended offering participants a range of flexible times to allow for environment choice, for example, fitting around family timetables [8,11]. Although this was not feasible in this study because the NGT and voting had to be conducted in real time, the modifications we have described contributed to ensuring participants felt at ease and promoted positive group dynamics [10,11].

### Limitations of the Evaluation

Although the response rate for the voting participants was acceptable, the majority of the open text comments came from one health care user (ie, HU2). The response rate for the study team was only 40% (4/10), with no responses received from the chairperson or observers. Furthermore, the COMET evaluation form was not tailored to the web-based meetings. To enrich the formative feedback and enhance the credibility of the present findings, the lead author (RK) sought an opportunity to triangulate our findings with 13 independent experts with experience in the planning and delivery of web-based consensus meetings. A meeting was convened in February 2021 by the Medical Research Council-National Institute for Health Research (NIHR) Trials Methodology Research Partnership Outcomes Working Group COS-subgroup, and experts joined virtually from the United Kingdom, Ireland, Amsterdam, the United States, Canada, and Australia. Agreed recommendations were directly relevant to many of our feedback findings, including the need for careful premeeting preparation, setting expectations to achieve less than what would be possible face-to-face, considering equity of engagement, ensuring the chairperson is strict with timings, and allowing time at the end for debriefing and reflection [21].

### Conclusions

The COVID-19 pandemic presented a need and opportunity to introduce and evaluate a web-based consensus method involving hearing-impaired participants. Our findings indicate that it is feasible to conduct successful web-based consensus exercises with multistakeholder groups using audiovisual virtual meeting technology. We anticipate that the methodological changes made and the lessons learned are more widely applicable to other forms of research that require consensus-based decision-making and are not necessarily limited to COS development studies.

## Appendix

Multimedia Appendix 1 Core Rehabilitation Outcome Set for Single-Sided Deafness study facilitator pack.

Multimedia Appendix 2 Core Rehabilitation Outcome Set for Single-Sided Deafness participants plan and guide documents for virtual consensus meetings.

Multimedia Appendix 3 Core Rehabilitation Outcome Set for Single-Sided Deafness preconsensus meeting introductory presentation slides.

Multimedia Appendix 4 Core Rehabilitation Outcome Set for Single-Sided Deafness preconsensus meeting introductory presentation recording.

Multimedia Appendix 5 Core Rehabilitation Outcome Set for Single-Sided Deafness consensus meeting evaluation form.

## Abbreviations

COMET: Core Outcome Measures in Effectiveness Trials
COS: core outcome set
CROSSSD: Core Rehabilitation Outcome Set for Single-Sided Deafness
NGT: nominal group technique
NIHR: National Institute for Health Research
SSD: single-sided deafness
