# Supplementary material for: Redesigning a Web-Based Stakeholder Consensus Meeting About Core Outcomes for Clinical Trials: Formative Feedback Study
Source: JMIR Form Res. 2021 Aug 19;5(8):e28878. doi: 10.2196/28878 (PMC8414289; doi:10.2196/28878)
Supplement: Multimedia Appendix 1 [file formative_v5i8e28878_app1.pdf]

Core Rehabilitation Outcome Set for Single Sided Deafness (CROSSSD) Study
Virtual Consensus Meeting to agree a Core Outcome Set (COS) for Single Sided Deafness (SSD) Interventions

Aim:
To bring together a sample of healthcare users and healthcare professionals who have completed both Rounds of the CROSSSD Study e-Delphi survey to discuss and agree on the final set of outcome domains for SSD interventions.

Date:
Tuesday 7th of July 2020, 9am-5pm.

Platform:
Microsoft Teams.

Study Management Team:
Roulla Katiri, PhD Student CROSSSD Study.
Deborah Hall, Professor of Hearing Sciences & PhD student supervisor.
Pádraig Kitterick, Associate Professor in Hearing Sciences & CROSSSD study chief investigator.

Facilitators:
Derek Hoare, Associate Professor in Hearing Sciences.
Kathryn Fackrell, NIHR Post-Doctoral Research Fellow (Hyperacusis).
Deborah Hall, Professor of Hearing Sciences.

Technical support:
Roulla Katiri, Participant training on online platform, set-up of meeting invites and address technical issues.
Pádraig Kitterick, Uploading and amendments of voting forms, assist with technical issues during the day.

Patient & Public Involvement (PPI) team:
Adele Horobin, Patient & Public Involvement manager NIHR Nottingham Hearing BRC.
Nora Buggy, PPI collaborator and healthcare user with SSD using a CROS aid.
Nicholas (Nicky) Hogan, PPI collaborator and healthcare user with SSD using a BAHA.
PPIs are allowed to participate in discussions throughout the day but *cannot* vote.

Participants:
Stakeholder representatives: healthcare users, healthcare professionals (audiologists and ENT surgeons), clinical researchers and commercial representatives.
Observers: Two commercial representatives (Oticon Medical); and a healthcare professional and clinical researcher.
Observers are *not* allowed to participate in discussions *nor* vote.

SSD, impact and interventions:
SSD: Normal or near-normal hearing in one ear and a severe to profound hearing impairment in the other ear.
Impact: Most significant functional consequences include difficulties understanding speech in noisy environments and locating where sounds are coming from.
'Rerouting' Interventions: Contralateral Routing of Signals (CROS) and Bone Anchored Hearing Aid (BAHA) which transfer signals from the poor side to the better hearing ear.
'Restoring' interventions: Cochlear Implants (CI) and Middle Ear Implants which get implanted on the poor side.
Historically CI was first implanted to address incapacitating tinnitus following sudden-onset SSD.

Participant sub-groups:

Groups and facilitators	Group A<br>Derek Hoare	Group B<br>Kathryn Fackrell	Group C<br>Deborah Hall
Study team helpers	Roulla	Pádraig	Adele
PPIs	Nicky	Nora	Adele
Healthcare users	Carly Sygrove<br>Richard Bowles	Chris Parker<br>Peter Toth	Lewis Williams<br>Roger Bayston
Healthcare professionals	Penny Feltham	Richard Nicholson	Paul James
Commercial representative	N/A	Cherith Campbell-Bell	Paddy Boyle
Clinical researchers	Ad Snik	N/A	N/A
Observer	Tove Rosenbom	Daniel Zeitler	Maxine Oxford

Participant expertise:
Carly Sygrove, Madrid. SSNHL, trialed CROS, no benefit. Blogged during recruitment https://bit.ly/2OFCubY.
Richard Bowles, Kent. SSD due to neuroma. CROS user.
Chris Parker, Preston. SSD due to neuroma. CROS user.
Peter Toth, Surrey. Congenital SSD. CROS user. Keen musician.
Lewis Williams, London. SSD due to labyrinthitis, implanted with CI in Berlin to address tinnitus.
Roger Bayston, Nottingham. CROS trial in the past but not using any intervention now. Prof of surgical infection, UoN.
Penny Feltham, Manchester Audiologist. PhD. Works in BAHA/CI. Member www.auronet.org/.
Richard Nicholson, Nottingham Audiologist. Expert on CROS aids.
Paul James, Berlin Audiologist. Involved in Lewis's CI implantation. Used to work at RNTNE.
Cherith Campbell-Bell, Cochlear commercial representative.
Paddy Boyle, Advanced Bionics commercial representative.
Ad Snik, Nijmegen Audiologist & Clinical researcher. Renowned in the SSD field www.snikimplants.nl.
Daniel Zeitler, Seattle, Virginia Mason, ENT & Clinical researcher https://bit.ly/31dITCc.
Tove Rosenbom, Smørum. Oticon Medical Denmark Senior Director Clinical Audiology and Research, BAHS.
Maxine Oxford, Oxford. Oticon Medical UK Sales and Marketing Director.

Social Media:
If you'd like to Tweet about the consensus meeting you can use the following tags:

Study UoN team	@CROSSSD_ @RouKat @padraig_hearing @HorobinAdele @Derek_J_Hoare @FackrellKathryn @DebHallNBRUH
Study organisation	@hearingnihr @UoNHearSci @NottsSPHL
Other related	@NIHRresearch @NIHRtakepart @NIHRinvolvement @Sharebank1 @COMETinitiative @COMITIDStudy @GlobalPPINet
Participants	@myhearingloss @PennyF_UK @radboudumc @DrDanielZeitler
Commercial reps	@oxford_maxine @OticonMedical @AdvancedBionics @CochlearUK @phonak
Charities	@BANAUK @MenieresSociety @ActionOnHearing @BritishTinnitus
Professional bodies	@BSAudiology1 @ENTUKGlobal @BCIG_UK @ENTANewsround
Steering group	@Prof_IainBruce @MFT_Research @Pvandeheyning @UAntwerpen @WUSTL_ENT

Please do not upload photos unless you obtain consent from the participants in your group to do so on the day.

Introductory presentation:
An introductory pre-recorded presentation was emailed to all participants, observers and PPI team on 27/06/2020. It covers the scope of the meeting, aims and objectives as well as the plan of the meeting. It will also provide details on COS development, including the results of the e-Delphi rounds. They were asked to watch the presentation prior to the meeting and email queries or questions to Roulla. Individual queries will be addressed by email and will also be mentioned on the day of the consensus meeting for the benefit of other attendees, as appropriate.

Consent forms:
All participants were asked to complete the consent form electronically after they watch the introductory presentation. They are able to fill this in online via O365 Forms. The consent form states that the discussions will be recorded. Roulla will ensure everyone has submitted their signed consent form by Monday 6th of July.

Outcome domains:
A total of 44 domains were chosen and defined at a workshop with Pádraig, Deb, Roulla, Adele, Nora and Nicky in Dublin (July 2019). An additional 5 domains were added to Round 2 of the e-Delphi following analysis of Round 1 participant suggestions. A total of 49 outcome domains were rated during Round 2 e-Delphi.

Rating scale and rules for outcome domain inclusion / exclusion:

1	2	3	4	5	6	7	8	9
Not at all important			***Important but not critical***			***Critical***		

- **IN** unless voted out on day: Outcomes where at least 70% of participants scored 7-9 and less than 15% scored 1-3, in all stakeholder groups.
- **OUT** unless voted in on the day: Outcomes where at least 50% of participants scored 7-9 in more than 1 stakeholder group.
- **MAYBE OUT** unless voted in on the day: Outcomes where less than 50% of participants scored 7-9 in all stakeholder groups.

A total of 14 domains were voted IN by at least 70% of the stakeholders in all stakeholder groups.

Domains (n=14) that fitted the INclusion rule: -the 'clear winners', should definitely always be included in a COS

**Domain category**	**Outcome domain**	**Outcome domain definition**
Other effects	**7. Listening effort**	Exerting greater effort to listen and follow a conversation. This might consequently lead to feelings of tiredness and fatigue; but those feelings would be a separate outcome domain
Factors related to the treatment being tested	**8. Treatment satisfaction**	How the treatment meets your expectations or how pleased you are after receiving the treatment; or how likely you are to recommend the treatment
	**9. Device usage**	How you use the device (for example; in what situations; for how long)
	**10. Device malfunction**	The device does not work as it should or it stops working
Health-related quality of life	**12.Avoiding social situations**	Choosing not to go to particular social situations because of your hearing loss
	**15. Impact on social situations**	Your hearing loss or device limiting your ability to fully participate in the social world; especially in challenging situations or where a lot of effort is needed to follow the conversation (for example; at a restaurant; at the park; in a bar or at a party)
	**16. Impact on work**	Effect of your hearing loss or device on your ability to carry out work tasks or job roles; or advancing your career
Hearing disability	**17. Being aware of a sound**	Being aware of a sound and recognising what that sound is (for example; being aware that someone has started to speak)
	**18. Listening in complex situations**	The difficulty experienced when listening to a sound while separating it out from a background of other sounds
	**19. Listening in reverberant conditions**	The difficulty experienced when listening in places where the sound reflects off the walls; floor or ceiling (echoes); creating a blurred sound. For example; understanding announcements in train stations or airports
	**21. Group conversation in quiet**	Listening and following a conversation between a group of people; in a quiet environment
	**22. One-to-one conversation in general noise**	Listening and understanding one person; in a noisy environment
	**23. Group conversation in noisy social situations**	Listening and following a conversation between a group of people; when others are talking in the background

Spatial hearing	**24. Sound localisation**	Knowing where a sound is coming from
	**26. Spatial orientation**	Knowing where you are in relation to the position of a sound source
Physical effects	**28. Physical tiredness**	Tiredness or fatigue from the effort of listening or when you need to turn your head repeatedly to listen in social situations
Self	**35. Personal safety**	How your hearing loss effects your awareness of potential hazards and threats in your daily life (for example; moving traffic; hazards at the workplace) and those you may not be able to see or hear (for example; other people behind you)

Domains (n=8) that didn't fit inclusion nor exclusion rules: -'maybes' or undecided, important but not essential in a COS

**Domain category**	**Outcome domain**	**Outcome domain definition**
Psychological effects	**6. Dissatisfaction with life**	Being unhappy because you feel you should be achieving or should have achieved more in your life
Physical effects	**30. Manual dexterity**	Having the fine motor skills needed to use your device effectively (for example; putting the device on; changing the batteries)
	**31. Tinnitus-related brain changes**	Changes in brain structure or function associated with tinnitus
	**32. Hearing-related brain changes**	Changes in brain structure or function associated with hearing loss
Self	**33. Self-stigma**	Negative perception of yourself due to your hearing loss and feeling stigmatised for using a hearing aid
Tinnitus	**40. Tinnitus awareness**	Noticing the sound of tinnitus is there
	**43. Tinnitus pitch**	Whether your tinnitus has a note-like quality; for example high pitch like whistling or low pitch like humming
	**44. Tinnitus quality**	What type of sound is heard (for example; hissing; buzzing; ringing; whistling etc)

Domains (n=19) that fitted the EXclusion rule: -the 'clear losers'

**Domain category**	**Outcome**	**Outcome definition**
Psychological effects	**2. Discomfort in listening situations**	Finding yourself in listening situations that you feel you can't adequately control (for example; when you can't choose a favourable listening position); or situations in which you don't feel comfortable (for example when interacting with people who don't know you have a hearing loss)
	**3. Emotional distress**	A negative unpleasant emotional reaction which may include fear; anger; frustration; anxiety; and suffering
	**4. Mood**	General sense of well-being; ranging from feeling very low or negative to very positive
	**5. Motivation**	A willingness to engage in challenging listening situations
Factors related to the treatment being tested	**11. Adverse events**	Any bad or unexpected thing that happens during the time a treatment is being tested in a clinical trial
Health-related quality of life	**13. Impact on individual activities**	Effect of your hearing loss or your device on your choice to engage in individual activities (for example; travelling alone; swimming or watching TV / films / movies)
	**14. Impact on relationships**	Effect of your hearing loss or your device on making new relationships and maintaining relationships with a spouse or partner; family; friends and colleagues
Spatial hearing	**25. Sound distance**	Knowing if a sound is close by or far away
Self	**35. Self-Image**	Feeling incomplete or incapable because you are unable to do all the things that you want to do
Sound quality	**37. Loudness**	How 'loud' a sound seems to you
	**38. Fullness**	How 'full' a sound seems to you. This can also be described as the 'richness'; 'warmth' or 'depth' of a sound

	**39. Clarity**	How 'clear' a sound seems to you
Tinnitus	**41. Tinnitus intrusiveness**	Being acutely aware of the sounds of tinnitus; feeling that it is invading your life or your personal space; changing your thoughts or actions and negatively impacting on your life
	**42. Tinnitus loudness**	How loud your tinnitus sounds
Factors related to the treatment being tested	**45. Device usability**	How easy it is to learn; use; and maintain the device (for example; changing the batteries; cleaning)
Health-related quality of life	**46. Impact on learning**	Effect of your hearing loss or device on your ability to acquire new knowledge or skills; or further your education
Psychological effects	**47. Independence**	How your hearing loss affects how much you need to rely on other people in daily life
Self	**48. Concern about your hearing**	Feeling worried about the hearing in your better ear and the thought that it may decline
	**49. Valnerability**	Feeling insecure because your hearing loss affects your awareness of potential hazards and threats in your daily life (for example; moving traffic; hazards at the workplace) and those you may not be able to see or hear (for example; other people behind you)

N.B. Dilemma: what we do with the small number of commercial representatives (n=7) results. The key concern was that some of the rules for considering outcome domains at the consensus meeting require consistency across all groups, and therefore the scores of a very small number of commercial reps could ultimately determine whether certain outcomes are even discussed (the maybes will be affected). See https://bit.ly/2NcrDVH.

'Top 3 Outcome Domains':
All participants will be asked to choose their Top 3 outcome domains and submit them by completing a short survey prior to the meeting. The results should be submitted by Friday 3rd of July and will be analysed; and will be uploaded in the CROSSSD Virtual Consensus Meeting folder in Teams, under Files.

Group discussions:
Roulla, Pádraig and Adele will help and support small sub-group discussions, monitor the chat and address participants' queries. If discussions are getting intense, then consider breaking the session for 5 minutes or so. Please be sure to ask the group 'have we missed anything?' before voting. Participants should feel free to ask questions, and no question is trivial.

Reaching an agreement:
Please ensure that members of all stakeholder groups play an equal role in prioritising the outcomes. Be sure to invite and listen to dissenting voices when agreeing the choices, but remember that agreement is ≥70% (not everyone has to agree!). Make sure you stick to the task and not get completely diverted by other issues that will have a space to be raised at other times in the meeting.

Communication between facilitators and study team:
Roulla will monitor the chat in the CROSSSD Virtual Consensus Meeting folder in Teams during the day. This is available to everyone in the study management team, the facilitators and the PPI manager. If any queries, questions or concerns are raised during the day please post them in the group chat. All relevant documentation can be found under 'Files' in the same folder.

Meeting links:
Everyone will be emailed meeting links to their calendar according to the group or sub-group they are meant to be participating in, according to the agenda. The introductory session Teams meeting link will be the same for all participants. The sub-group links will be different etc. It is therefore important that everyone keeps on time. If there is any concerns during the day re: timekeeping please inform the group in the study communication chat.

CROSSSD
CORE REHABILITATION OUTCOME SET for SINGLE SIDED DEAFNESS

Agenda and activities:

Time slot	Introductory Session	Tool	Session lead	Chat helper(s)
09:00-09:15	Facilitators & Study Team can join the meeting and ask any questions / clarify last-minute queries	Calendar Teams Link	Roulla	N/A
09:15-09:30	Participants arrival	Calendar Teams Link	Roulla	Adele
09:30-09:35	Welcome, quick reminder of aims and Q&A	Intro section of slide deck	Roulla	Deb / Pádraig
09:35-09:45	Speedy ice-breaker activity, 30s intro for all participants	Table with all participants names	Roulla	Deb / Pádraig
09:45-09:55	Practice vote 1: Q: What stakeholder Grp are you in? (A: 4 choice)	Survey link	Pádraig	Roulla / Deb
09:55-10:00	Presentation of Pre-Meeting survey 'Top 3' results: outcome domains that were not in any of the participants' 'Top 3' choices	Top 3 Survey results of slide deck	Roulla	Deb / Pádraig

Time slot	Session 1: Decide which domains should be discussed during the day	Tool	Session lead	Chat helper(s)
10:00-10:05	Advise that will break into 3 sub-groups for discussion of 'Top 3' vote: *'These outcome domains were not in anyone's 'Top 3', remove them from COS?'*	Individual group 'Join meeting' links in chat + Slide with Survey Results	Roulla	Adele
10:05-10:30	Break into 3 sub-groups to discuss<br>Group A: Nicky, Carly, Richard B, Penny, Ad, (Tove)<br>Group B: Nora, Chris, Peter, Richard N, Cherith, (Daniel)<br>Group C: Lewis, Roger, Paul, Paddy, (Maxine)	Slide with Survey Results + Chat helper's word document for notes	A: Derek<br>B: Kathryn<br>C: Deb	Take notes<br>A: Roulla<br>B: Pádraig<br>C: Adele
10:30-10:45	Return to large group, each group's facilitator summarises discussion outcomes in less than 5 min each	Chat helper's word document with notes	A: Derek<br>B: Kathryn<br>C: Deb	Roulla
10:45-11:00	**Group VOTE:** *Agree with plan to EXCLUDE those outcome domains that were not in anyone's 'Top 3'?*<br>-YES, agree<br>-NO, disagree<br>- Unsure<br>If ≥70% (i.e. 9 out of 12) participants vote YES, proceed with those XX domains only.	UoN Survey Link	Pádraig	Roulla / Deb
11:00	**Session 1 Close –Break** (and preparation for next session)		Roulla	Roulla

Time slot	Session 2: Round 1 of domain exclusion	Tool	Session lead	Chat helper(s)
11:30-11:35	Advise that will break into 3 sub-groups to discuss and shorten list of domains –?aim for Top 5	Slide with all remaining domains following previous session vote	Deb	Roulla
11:35-12:15	Break into 3 sub-groups to discuss<br>Group A: Nicky, Carly, Richard B, Penny, Ad, (Tove)<br>Group B: Nora, Chris, Peter, Richard N, Cherith, (Daniel)<br>Group C: Lewis, Roger, Paul, Paddy, (Maxine)<br>Discuss shortened list of domains: *'Clear winners (always include), clear losers (low priority, drop from COS) and maybes (undecided, important but not necessary for a COS)?'*	Individual group 'Join meeting' links in chat + Slide with the red string	A: Derek<br>B: Kathryn<br>C: Deb	Take notes<br>A: Roulla<br>B: Pádraig<br>C: Adele
12:15-12:30	Sub-groups return to large group and feedback on Group's ?Top 5 with reasons	Large group 'Join meeting' link in chat	A: Derek<br>B: Kathryn	Roulla

		+ Chat helper's word document for notes	C: Deb	
12:35-12:40	Summary of domains: *Clear winners*, *Clear losers* and *Maybes*	Summary slide with list of domains	Roulla	Pádraig
12:30-12:40	**Group VOTE:** *Agree with plan to EXCLUDE those outcome domains that were not in anyone group's 'Top 5' (clear losers)?*<br>-YES, agree<br>-NO, disagree<br>- Unsure<br>If ≥70% (i.e. 9 out of 12) participants vote YES, proceed with those XX included domains only.	UoN Survey Link	Pádraig	Roulla / Deb
12:45	**Session 2 Close –Lunch** (and analysis / re-grouping of domains)	Presentation slide summarizing small group outcomes		
	**Study team and facilitators:** Reflect and feedback on aspects that are not going to plan? / need to address during afternoon session	Teams CROSSSD group chat	Roulla / Pádraig / Deb	Adele / Derek / Kathryn (25min each)

Time slot	Session 3: Round 2 of domain exclusion	Tool	Session lead	Chat helper(s)
13:30-13:35	Presentation of list of outcomes needing further discussion	Presentation slide with *clear winners* and *maybes*	Roulla	
13:35-14:00	Break into 3 sub-groups to discuss<br>Group A: Nicky, Carly, Richard B, Penny, Ad, (Tove)<br>Group B: Nora, Chris, Peter, Richard N, Cherith, (Daniel)<br>Group C: Lewis, Roger, Paul, Paddy, (Maxine)<br>Discuss shortened list of domains: *'Clear winners (always include), clear losers (low priority, drop from COS) and maybes (undecided, important but not necessary for a COS)?'*	Individual group 'Join meeting' links in chat + Slide with the red string	A: Derek<br>B: Kathryn<br>C: Deb	Take notes<br>A: Roulla<br>B: Pádraig<br>C: Adele
14:00-14:15	Sub-groups return to large group and feedback on Group's ?Top 5 with reasons	Large group 'Join meeting' link in chat	A: Derek<br>B: Kathryn<br>C: Deb	Roulla
14:15-14:20	Summary of domains: *Clear winners*, *Clear losers* and *Maybes*	Summary slide with list of domains	Roulla	Pádraig
14:20-14:25	**Group VOTE:** *Agree with plan to EXCLUDE those outcome domains that were not in anyone group's 'Top 5'?*<br>-YES, agree<br>-NO, disagree<br>- Unsure<br>If ≥70% (i.e. 9 out of 12) participants vote YES, proceed with those XX included domains only.	UoN Survey Link	Pádraig	Roulla / Deb
14:30	**Session 3 Close –Break** (and preparation for next session)		Roulla	Roulla

Time slot	Session 4: Domain inclusion	Tool	Session lead	Chat helper(s)
15:00-15:05	Presentation of short list of remaining outcomes, the '*clear winners*'	Presentation slide with *clear winners* (and *Maybes?)*	Roulla	
15:05-15:15	**Group VOTE:** *Agree this list of outcome domains should always*	UoN Survey Link	Deb	Roulla

	be in a COS for SSD interventions?<br>-YES, agree<br>-NO, disagree<br>- Unsure<br>If ≥70% (i.e. 9 out of 12) participants vote YES, proceed with those XX included domains only.			
15:15-15:20	Presentation of *clear winners* to be included in the COS	Presentation slide	Deb	

Time slot	Session 5: Additional tasks if have time	Tool	Session lead	Chat helper(s)
15:30	Definitions of outcome domains?<br>Everyone agrees that included domains definitions are clear?	Presentation slide with definitions		
15:45	Discussion regarding prioritization of outcome domains?<br><br>Future research will be concentrating on instrument recommendations> which one is the best, there will be regardless > Q is where to start? Which one will be the Top 1 or 2 or 3	Slide with ranking of outcome domains?<br><br>*Draft of a survey on what they are ranking*		
16:00	Q&A<br>• Any other discussion points?<br>• Participant feedback they'd like to share?<br>• One positive aspect of the meeting / what to work on?	Can have a slide with bullet points, share screen 'take notes'?		
16:15	Thank you, Future Steps & Close	Presentation slides ?? / Reminder to complete evaluation form	Roulla	

Facilitators to have in mind for sub-group discussions:

- Use the presentation slide with the 17 Domains in Cards & 'Red String'.
- Discuss and jointly agree top 5 by moving the cards around to facilitate discussion (e.g. if put one in the top 5, need to take another out).
- Emphasise to the group that the more items considered to be important and critical at this stage, the less helpful this exercise will be in terms of guiding clinical trial designs.
- Participants should be encouraged to share their reasons for ranking near the top or bottom to help discussion.
- Discussion should relate to measuring the effect of the intervention in a **clinical trial**.
- Remind participants to focus on discussing 'the **WHAT'** should be included in COS. Don't worry about 'the HOW' it should be measured.
- Remind participants that the Top 5 list has got to include outcomes that are relevant to all interventions, and that will be most sensitive to change over the course of the treatment.
- Save your group's Top 5; the helper should make a note of any strong dissenting views or important comments using the word document for notes.

If we are going ahead with prioritization task:

- Explain why it's important to rank relative importance (To plan Phase 2 –the **HOW**).
- Start with those outcomes where ≥70% participants agree should be in the top 5. Remember that there may be fewer than 5 at this point. That's fine. 5 is only a maximum, not necessarily an absolute target.
- Arrange domains on the slide in an initial order according to the discussions so far??
- Try to achieve an ordered set (no ties allowed) through discussion and by moving the cards around.
- Once the group seem to have exhausted all discussion and there is an interim list, then cast this order to a vote?
