# Supplementary material for: Redesigning a Web-Based Stakeholder Consensus Meeting About Core Outcomes for Clinical Trials: Formative Feedback Study
Source: JMIR Form Res. 2021 Aug 19;5(8):e28878. doi: 10.2196/28878 (PMC8414289; doi:10.2196/28878)
Supplement: Multimedia Appendix 2 [file formative_v5i8e28878_app2.pdf]

Core Rehabilitation Outcome Set for Single Sided Deafness (CROSSSD) Study

Consensus meeting to agree a Core Outcome Set (COS) for Single Sided Deafness (SSD) Interventions

In preparation for the meeting, it would be helpful if you could read this document carefully.

Meeting focus:
The aim of the consensus meeting is to bring together a sample of healthcare users, healthcare professionals, clinical researchers and commercial representatives who have completed both Rounds of the e-Delphi survey; to discuss and agree the final minimum set of outcome domains for SSD interventions.

Date and time:
Tuesday 7th of July 2020, 9:30am to 4:30pm. Please log-into Microsoft Teams promptly just before 9:30am. We aim to wrap up the meeting by 4-4:30pm.

Virtual meeting platform:
We will be using Microsoft Teams for the meeting. You can join via the Teams App or on the web. To join simply click on the 'Join Microsoft Teams Meeting' link which will be send to your email calendar in due course. More details on the Teams software and on how to join can be found in this short video.

To improve the sound quality for all participants during the meeting please ensure your microphone is muted when you are not talking. It is preferable to have your camera turned on, to enhance communication for other participants who use lip-reading. Please 'raise your hand' and use the chat function to attract the facilitator's attention or post any comments or questions to the group. For details on how to use these features please refer to the diagram below, which will be on your screen. You can also enable captions on Microsoft Teams, if you feel that might be helpful. If you'd like any more details on these features please email roulla.katiri@nottingham.ac.uk.

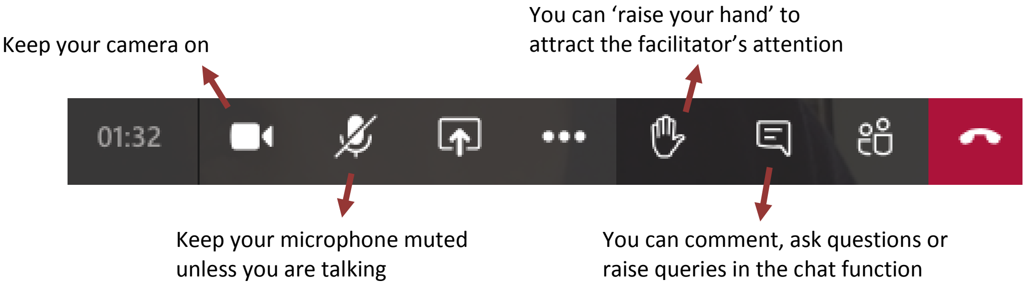

Study management team:
Roulla Katiri, PhD student CROSSSD study
Deborah Hall, Professor of Hearing Sciences, expert in outcome measures & PhD student supervisor
Pádraig Kitterick, Associate Professor in Hearing Sciences, expert in SSD & CROSSSD study chief investigator

Facilitators:
Derek Hoare, Associate Professor in Hearing Sciences
Kathryn Fackrell, NIHR Post-Doctoral Research Fellow (Hyperacusis)
Deborah Hall, Professor of Hearing Sciences.

Patient & Public Involvement (PPI) team:
Adele Horobin, Patient & Public Involvement manager NIHR Nottingham Hearing BRC
Nora Buggy, PPI collaborator and healthcare user with SSD using a CROS aid
Nicholas (Nicky) Hogan, PPI collaborator and healthcare user with SSD using a BAHA
PPIs are allowed to participate in discussions throughout the day but *cannot* vote.

Participants:
Stakeholder representatives: Expert healthcare users, healthcare professionals, clinical researchers and commercial representatives.
Observers: Two commercial representatives (Oticon Medical); and a healthcare professional and clinical researcher. Observers are *not* allowed to participate in discussions *nor* vote.

Participant sub-groups:
To help with discussions throughout the day, we have split the larger group into three sub-groups as listed on the table below. You will have separate Microsoft Teams links saved in your email calendar, directing you to the correct group at the correct time on the day. If you have any queries with regards to your allocated group please get in touch.

Groups and facilitators	**Group A**<br>Derek Hoare	**Group B**<br>Kathryn Fackrell	**Group C**<br>Deborah Hall
Study team helpers	Roulla Katiri	Pádraig Kitterick	Adele Horobin
Patient & Pubic Involvement (PPI) team	Nicky Hogan	Nora Buggy	Adele Horobin
Healthcare users<br>Healthcare professionals<br>Commercial representatives<br>Clinical researchers<br>Observers	Ad Snik<br>Carly Sygrove<br>Penny Feltham<br>Richard Bowles<br>Tove Rosenbom	Cherith Campbell-Bell<br>Chris Parker<br>Daniel Zeitler<br>Peter Toth<br>Richard Nicholson	Lewis Williams<br>Maxine Oxford<br>Paddy Boyle<br>Paul James<br>Roger Bayston

Draft Agenda (will be flexible during the day, will be guided by discussions)**:**
Time in GMT
09:15-09:30 Log-in & Join Microsoft Teams Meeting
09:30-10:00 Welcome, Introductions and Q&A
10:00-10:45 Group discussions on outcome domains to consider
10:45-11:15 *Coffee Break*
11:15-11:45 Small Group Workshop 1: Reducing the number of outcome domains for clinical trials
11:45-12:30 Workshop feedback to the larger group
12:30-13:00 *Lunch*
13:00-13:20 Small Group Workshop 2: Reducing the number of outcome domains for clinical trials
13:20-13:45 Workshop feedback to the larger group
13:45-14:15 *Coffee Break*
14:15-16:00 Discussion of workshop outcomes and agreement on final Core Outcome Set
16:00-16:30 Close & Feedback

What do I need to do on the day?
The study management team will briefly remind the group of the scope of the day and will answer any questions you may have. You will be expected to participate in small-group discussions to share your views; and voting exercises using Microsoft Forms. Links to these voting forms will be provided on the day of the meeting.

Preparation prior to the meeting:
To prepare for the meeting and to be aware of the Scope, Aims and Objectives please watch our 15 minute long introductory presentation.

The intention of the consensus meeting is to reduce the list of 17 outcome domains (see table below); but this will be confirmed with the group. These were scored as critical and important to include in a core outcome set for SSD interventions by at least 70% of the 241 participants, from 30 different countries, who took part in Round 2 of our online survey.

No	Outcome Domain	Outcome Domain Definition
7	Listening effort	Exerting greater effort to listen and follow a conversation. This might consequently lead to feelings of tiredness and fatigue; but those feelings would be a separate outcome domain
8	Treatment satisfaction	How the treatment meets your expectations or how pleased you are after receiving the treatment; or how likely you are to recommend the treatment
9	Device usage	How you use the device (for example; in what situations; for how long)
10	Device malfunction	The device does not work as it should or it stops working
12	Avoiding social situations	Choosing not to go to particular social situations because of your hearing loss
15	Impact on social situations	Your hearing loss or device limiting your ability to fully participate in the social world; especially in challenging situations or where a lot of effort is needed to follow the conversation (for example; at a restaurant; at the park; in a bar or at a party)
16	Impact on work	Effect of your hearing loss or device on your ability to carry out work tasks or job roles; or advancing your career
17	Being aware of a sound	Being aware of a sound and recognising what that sound is (for example; being aware that someone has started to speak)
18	Listening in complex situations	The difficulty experienced when listening to a sound while separating it out from a background of other sounds
19	Listening in reverberant conditions	The difficulty experienced when listening in places where the sound reflects off the walls; floor or ceiling (echoes); creating a blurred sound. For example; understanding announcements in train stations or airports
21	Group conversation in quiet	Listening and following a conversation between a group of people; in a quiet environment
22	One-to-one conversation in general noise	Listening and understanding one person; in a noisy environment
23	Group conversation in noisy social situations	Listening and following a conversation between a group of people; when others are talking in the background
24	Sound localisation	Knowing where a sound is coming from
26	Spatial orientation	Knowing where you are in relation to the position of a sound source
28	Physical tiredness	Tiredness or fatigue from the effort of listening or when you need to turn your head repeatedly to listen in social situations
35	Personal safety	How your hearing loss effects your awareness of potential hazards and threats in your daily life (for example; moving traffic; hazards at the workplace) and those you may not be able to see or hear (for example; other people behind you)

Ahead of the meeting you are required to consider your **'Top 3'** most important and critical domains to include in the core outcome set **from your own personal perspective** (as a healthcare user, healthcare professional, clinical researcher or commercial representative). Please remember that the core outcome set will be a recommendation to always measure *as a minimum* in all *clinical trials* that investigate SSD interventions in adults. At this stage of the process we are not concerned about how easy, time-consuming, fun, challenging or complicated it might be to measure this outcome; that is work for the future.
*Please submit your 'Top 3' outcome domains by **Friday 3rd of July** by completing this short survey.

Consent:
In order to take part you need to read and complete the study consent form, which can be accessed here. Please complete the consent form by **Friday 3rd of July**. If you need more information on the study prior to signing the consent form please read our information leaflets; for healthcare users and for professionals accordingly. Please contact Roulla Katiri at roulla.katiri@nottingham.ac.uk if you have any questions. Meeting observers do not need to complete the consent form.

Recordings:
To facilitate analysis, discussions will be recorded on Microsoft Teams. The recordings will be saved on University of Nottingham secure servers. In case they need to be transcribed, only non-identifiable information will be transcribed e.g. the identity of any of the talkers or anything said that identifies an individual will be removed.

Photography and Social Media:
The study team plan to take screen shots on the day, which will be shared on the internet and social media. If you do not wish to be included on social media posts, please let us know in advance. If you'd like to Tweet about the consensus meeting you may like to use the following tags:

Study UoN team	@CROSSSD_ @RouKat @padraig_hearing @HorobinAdele @Derek_J_Hoare @FackrellKathryn @DebHallNBRUH
Study organisation	@hearingnihr @UoNHearSci @NottsSPHL
Other related	@NIHRresearch @NIHRtakepart @NIHRinvolvement @Sharebank1 @COMETinitiative @COMITIDStudy @GlobalPPINet
Participants	@myhearingloss @PennyF_UK @radboudumc @DrDanielZeitler
Commercial reps	@oxford_maxine @OticonMedical @AdvancedBionics @CochlearUK @phonak
Charities	@BANAUK @MenieresSociety @ActionOnHearing @BritishTinnitus
Professional bodies	@BSAudiology1 @ENTUKGlobal @BCIG_UK @ENTANewsround

Meeting evaluation:
We would like to evaluate your experience of taking part in a virtual consensus meeting using an evaluation form. It is anticipated that completion of this will take approximately 5 minutes and completion is entirely voluntary.

Study contact:
Please get in touch with Roulla Katiri if any queries arise prior to the meeting or on the day.
Email: roulla.katiri@nottingham.ac.uk **Phone, WhatsApp or Viber:** +353 86 8966461 **Skype:** @roullak

Thank you for taking an interest in the CROSSSD study.
