# Supplementary material for: Redesigning a Web-Based Stakeholder Consensus Meeting About Core Outcomes for Clinical Trials: Formative Feedback Study
Source: JMIR Form Res. 2021 Aug 19;5(8):e28878. doi: 10.2196/28878 (PMC8414289; doi:10.2196/28878)
Supplement: Multimedia Appendix 3 [file formative_v5i8e28878_app3.pdf]

Core **R**ehabilitation **O**utcome **S**et for **S**ingle-**S**ided **D**eafness (CROSSSD) Study

CROSSSD

CORE REHABILITATION OUTCOME SET for SINGLE SIDED DEAFNESS

Virtual consensus meeting to agree a **Core Outcome Set** for single-sided deafness interventions

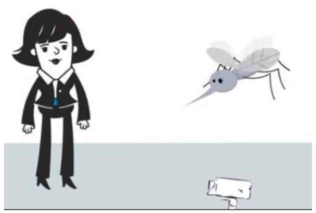

Tuesday 7th of July 2020,

Microsoft Teams

Thank You!

Study Management Team

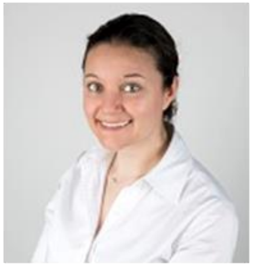

Roulla Katiri,
PhD Student

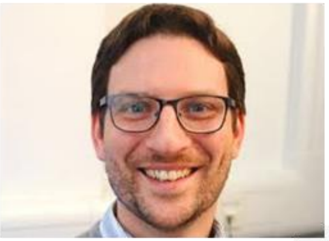

Pádraig Kitterick,
SSD Expertise

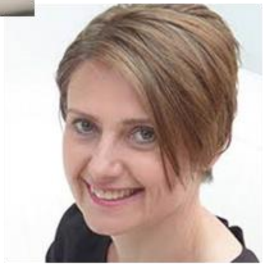

Deborah Hall,
Expertise in
Outcome Measures

Steering Group

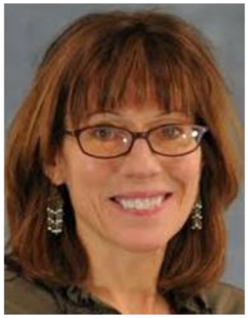

Jill B. Firszt,
St Louis, USA

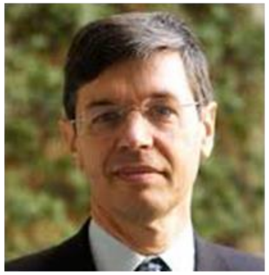

Paul Van de Heyning,
Antwerp, EU

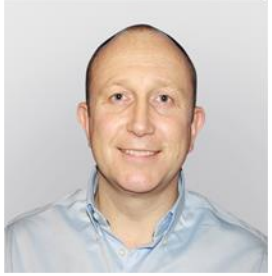

Iain Bruce,
Manchester, UK

Patient & Public Involvement

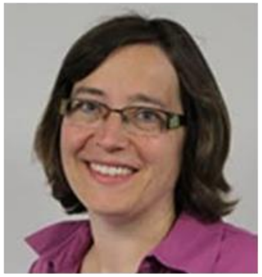

Adele Horobin,
PPI Manager

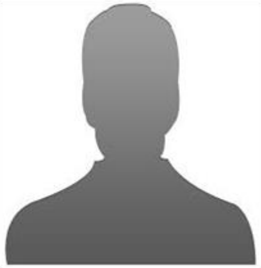

CROS
PPI Collaborator

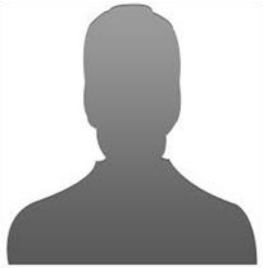

BAHA
PPI Collaborator

Why do we need a Core Outcome Set (COS) for Single-Sided Deafness (SSD) interventions?

Available SSD interventions

CROSSSD

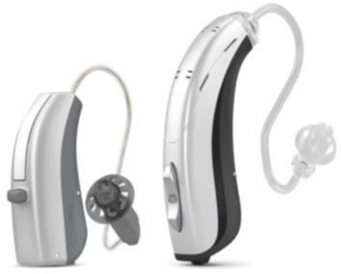

Contralateral Routing of Signals (CROS) Hearing Aid

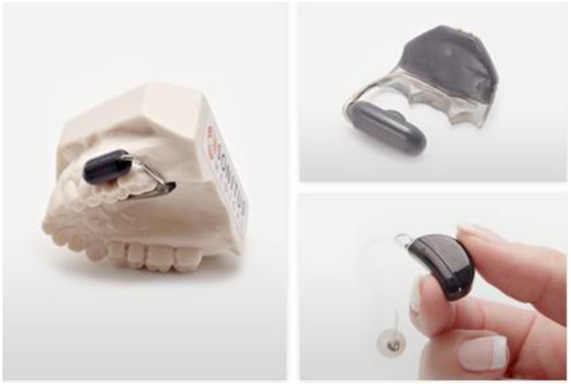

SoundBite™

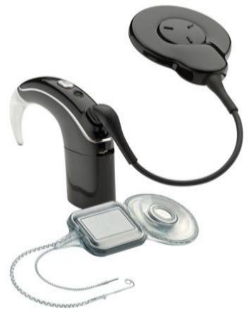

Cochlear Implant

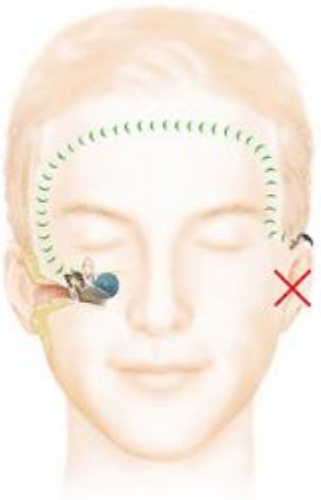

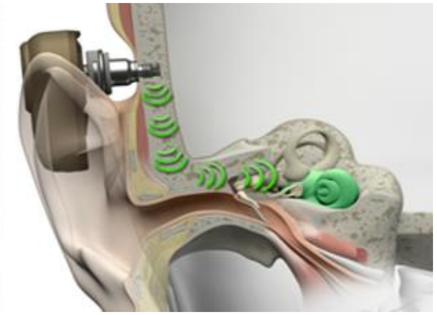

Bone Anchored Hearing Aid (BAHA)

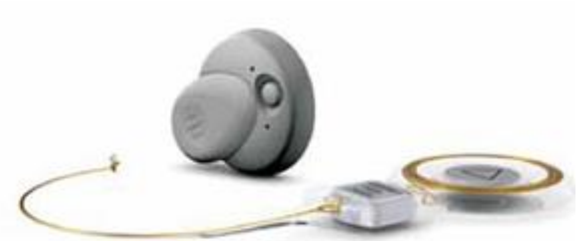

Middle Ear Implant

What is the evidence?

Speech Perception in Quiet / Noise

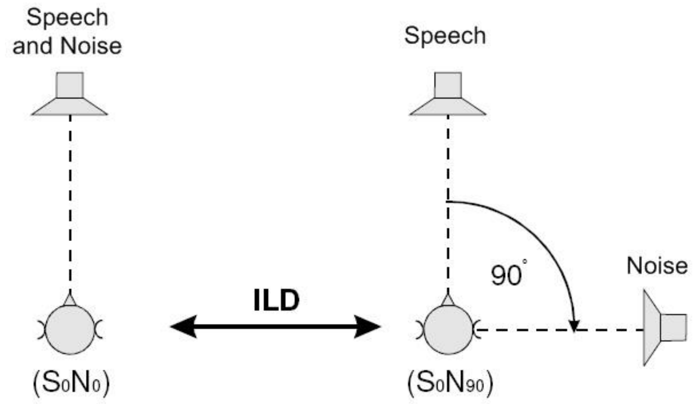

Electroacoustic Pitch Matching

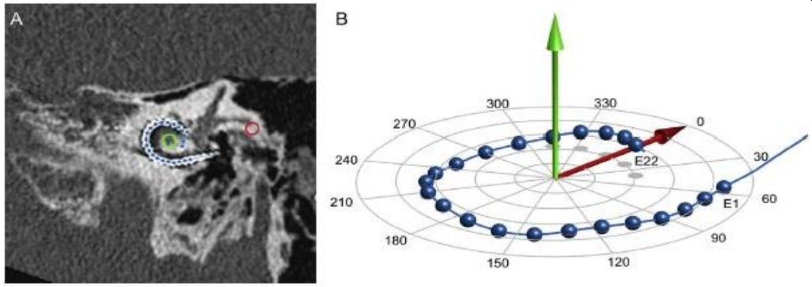

Quality of Life

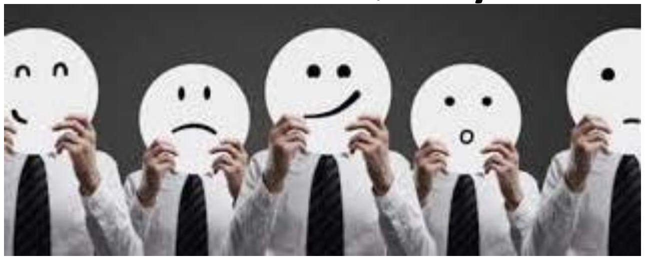

Neural substrates / Improvement of Tinnitus

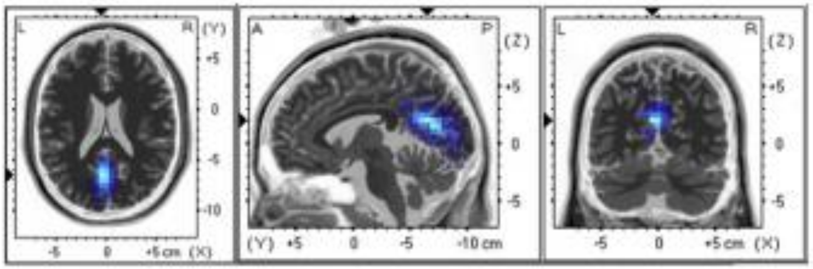

Sound Localisation Accuracy & Speech Understanding

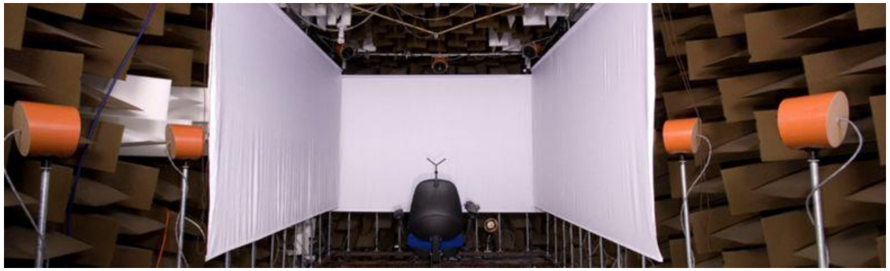

What is the problem?

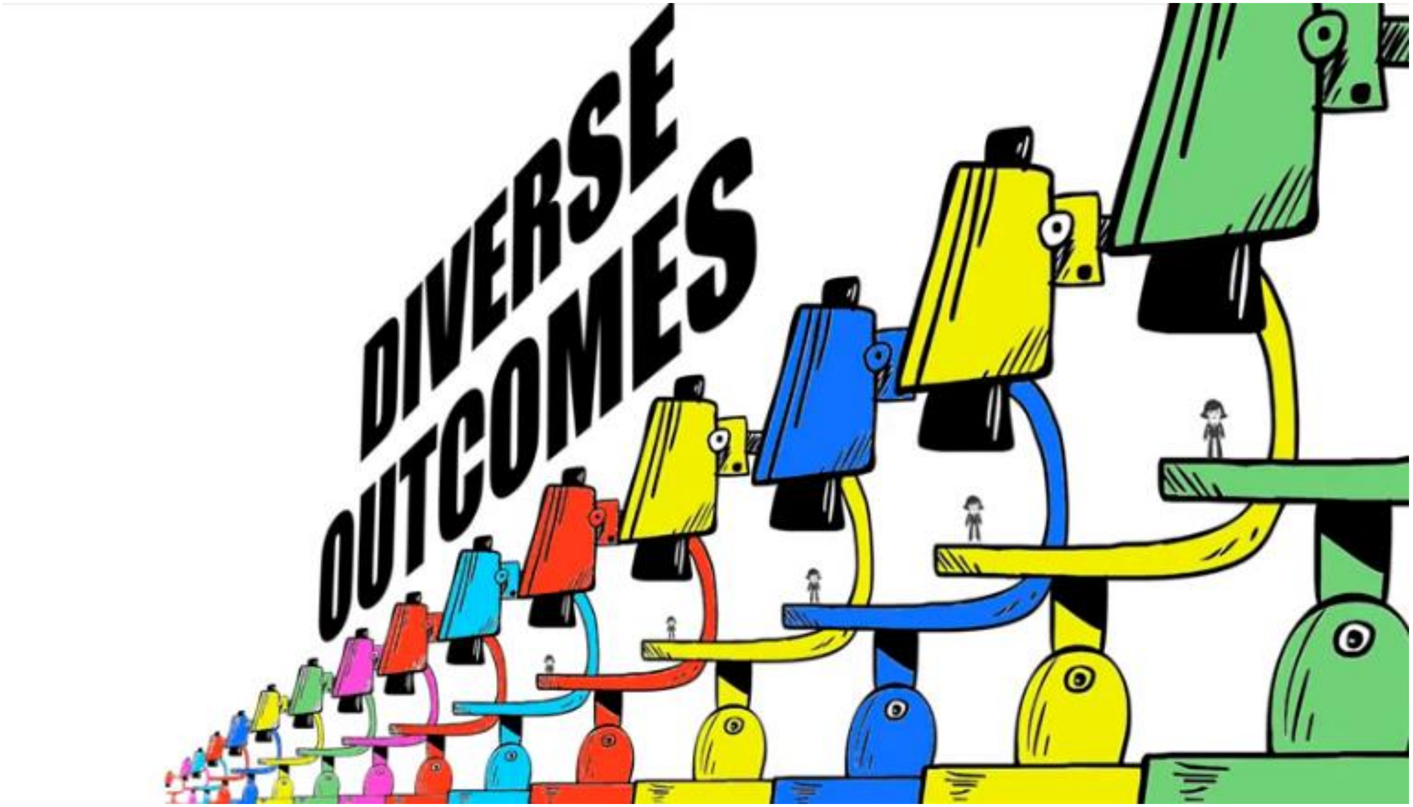

Example Trial A

Healthcare users with SSD

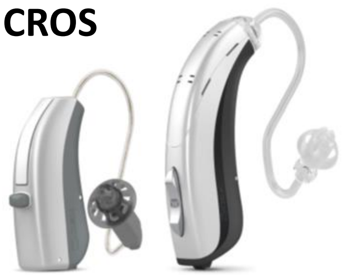

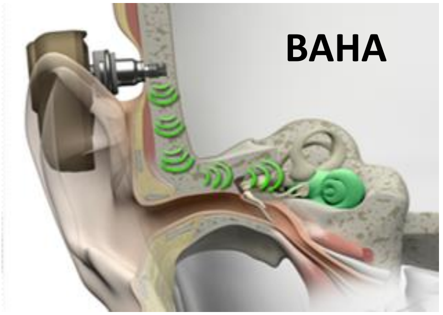

Outcomes used:

- Speech perception in noise (sentences)
- Speech perception in noise (single words)
- Hearing disability questionnaire

Example Trial B

CROSSSD

Healthcare users with SSD

CROS

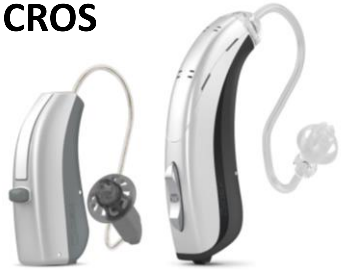

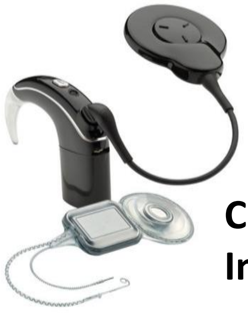

Cochlear Implant

Outcomes used:

- Quality of life
- Speech perception in noise (dichotic hearing)
- Auditory perception
- Sound localisation

Example Trial C

Healthcare users with SSD

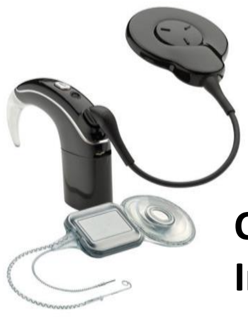

Cochlear Implant

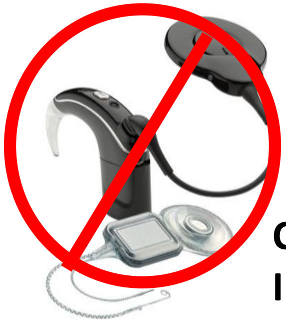

Cochlear Implant OFF

Outcomes used:

Speech perception in noise (test not stated)

Tinnitus loudness

CROSSSD

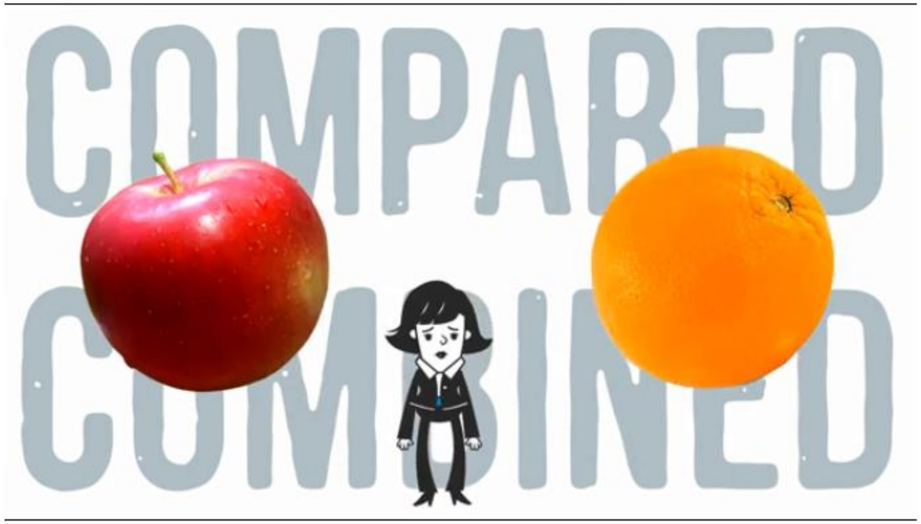

Evidence-based clinical care and decision making is currently limited by the use of multiple, heterogenenous, unvalidated outcome measures

What are Core Outcome Sets?

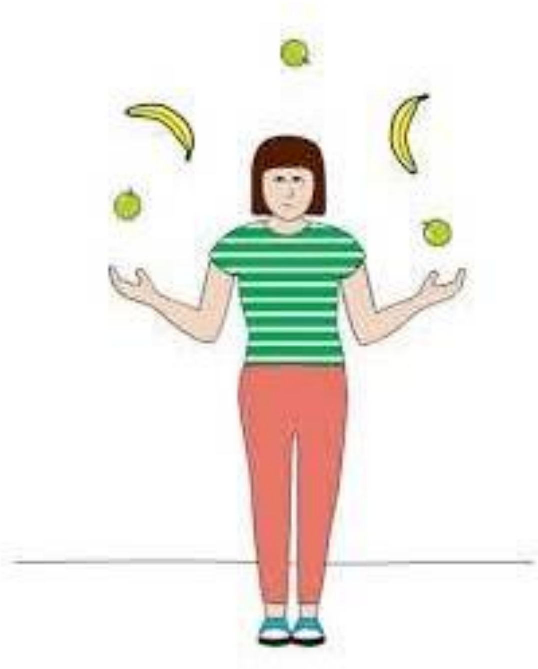

https://youtu.be/g1MZi2mzK1U

NIHR | National Institute for Health Research

CROSSSD Study:

Identifying what is critical and important to measure when evaluating hearing interventions for adults with Single-Sided Deafness (SSD)

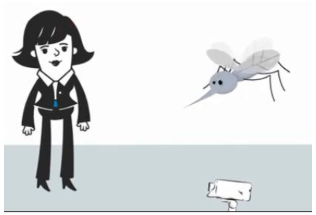

Consensus meeting aim

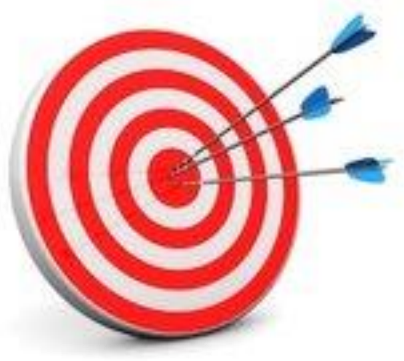

To finalise a list of outcomes which should be measured and reported, <u>*as a minimum*</u>, by researchers studying SSD interventions

CROSSSD
CORE REHABILITATION OUTCOME SET for SINGLE SIDED DEAFNESS

‘Core Outcome Set for SSD’

Outcome 1

Outcome 2

Outcome 3

Outcome 4

Outcome 5

Outcome 6

+

Any other additional outcomes the clinical trial team would like to measure

- Outcome 7
- Outcome 8
- Outcome 9 etc

Meeting goal

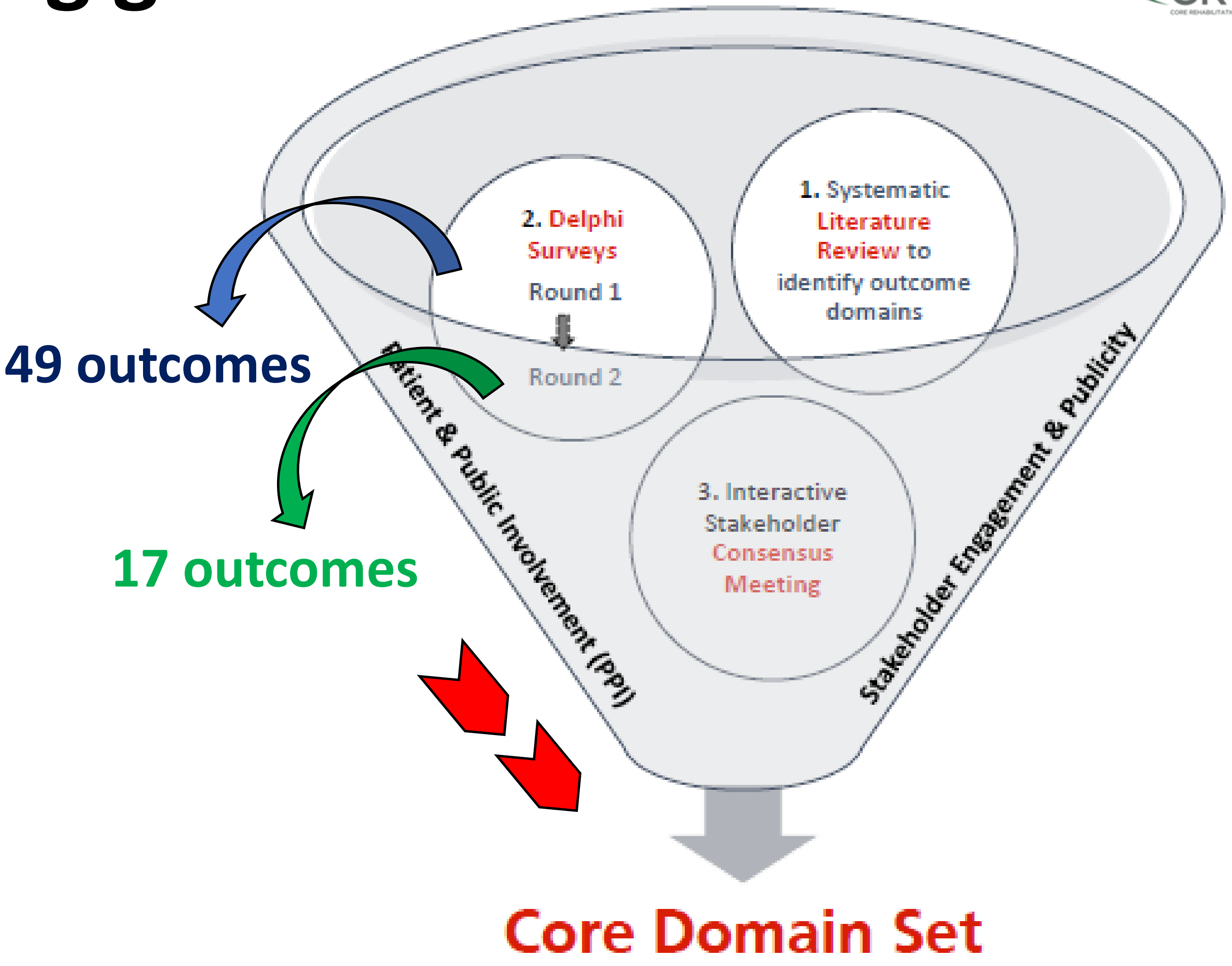

Next Steps

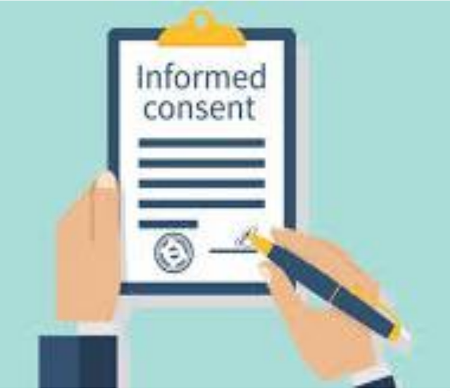

Complete the online consent form by
Friday 3rd of July
(Check your email for link to the form)

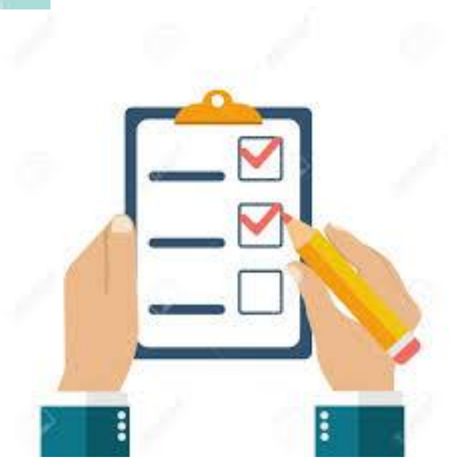

Identify your Top 3 Outcome Domains
by Friday 3rd of July
(Check your email for survey link)

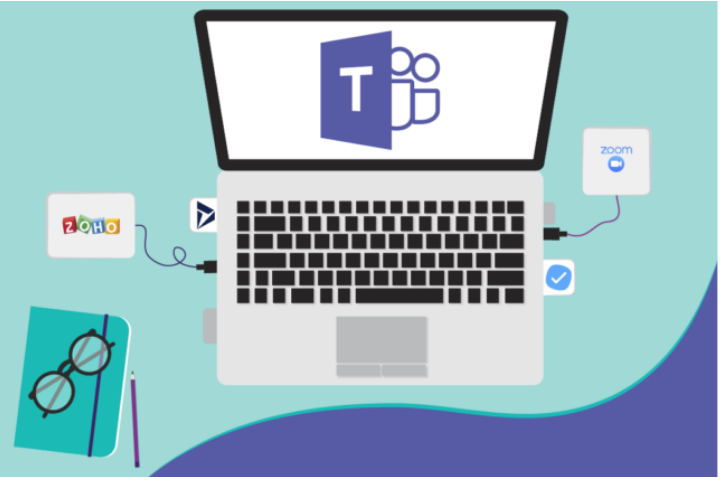

Group discussions and voting
on Tuesday 7th of July

Remember

CROSSSD
CORE REHABILITATION OUTCOME SET for SINGLE SIDED DEAFNESS

- The derived COS is for *clinical trials* not clinical use
- We are concerned about ***the 'WHAT'*** to measure
- The 'HOW' to measure each outcome is work for the future
- All opinions matter, please share them, we are all equal in this meeting despite what group we represent

Virtual 'Housekeeping'

- Sharp 9:30am start (GMT)
- Click on the 'Join Microsoft Teams Meeting' link in your calendar
- Breaks & Lunch
- Language use
- Mute your microphone
- Social Media
- Confidentiality
- Acknowledgements

Don't forget...

CROSSSD

Optional pre-consensus meeting coffee morning...
(Bring your own refreshment)

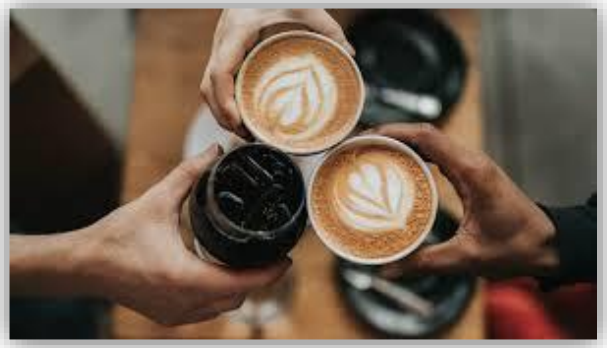

Thursday **2nd of July**
join any time 9:30-11:30am

- Test the technology
- Meet the other participants

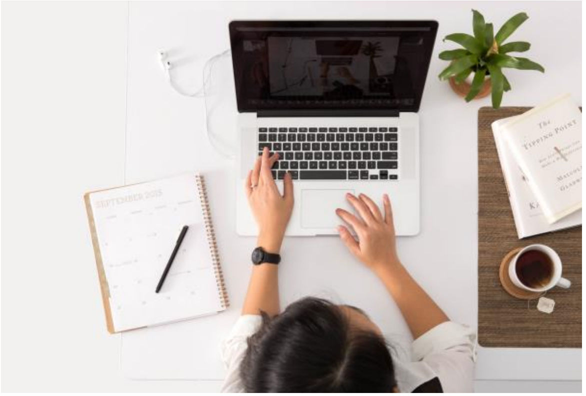

(Check your calendar for the
Join Microsoft Teams Meeting link)

Questions?

CROSSSD

To ensure we address all questions effectively please email them to roulla.katiri@nottingham.ac.uk by Monday 6th of July 2020

List of the 17 outcome domains that were unanimously voted, by all stakeholder groups, as critical and important to be included in a core domain set for SSD

7. LISTENING EFFORT

Exerting greater effort to listen and follow a conversation.
This might consequently lead to feelings of tiredness and fatigue; but those feelings would be a separate outcome domain

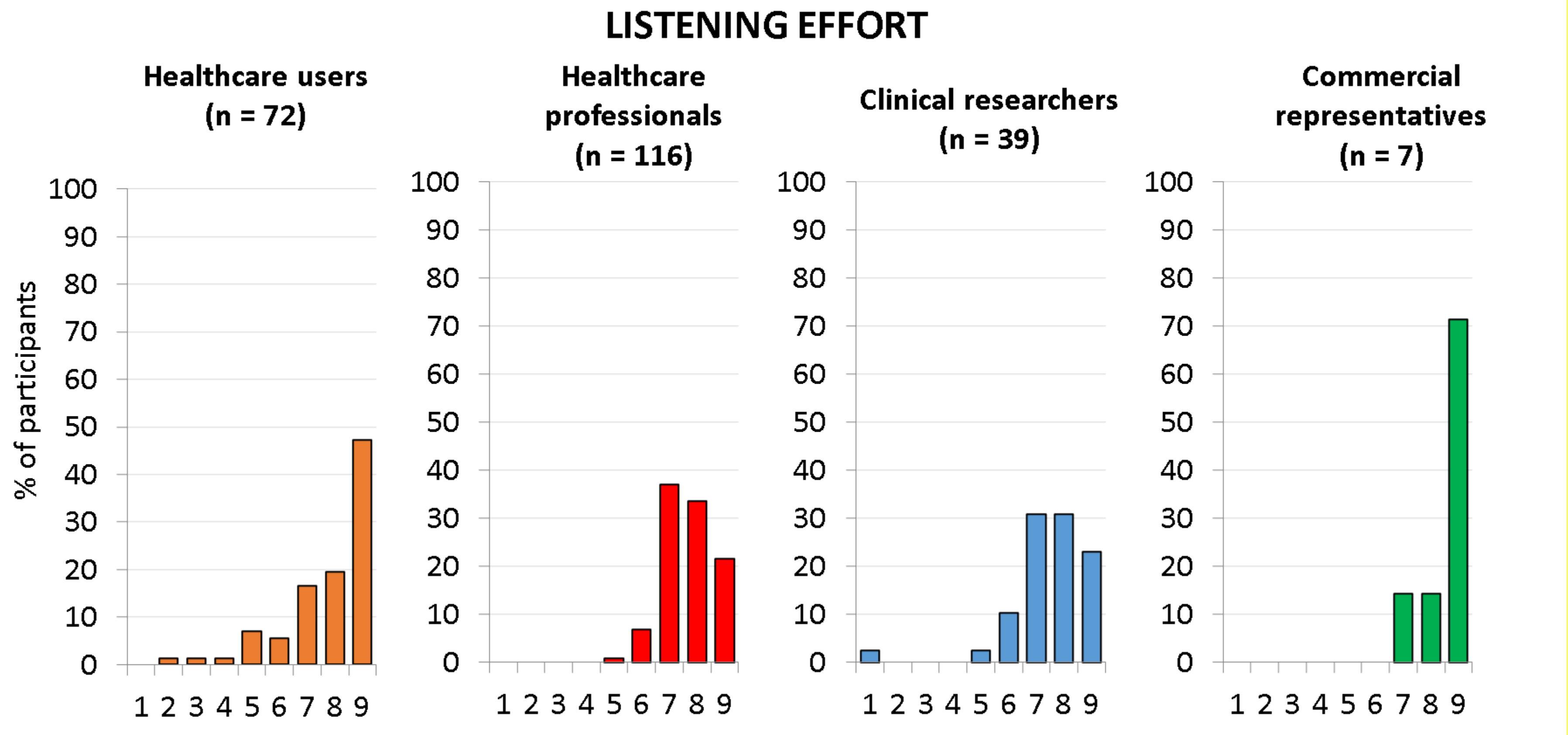

Domain Category:
Other effects

NIHR National Institute for Health Research

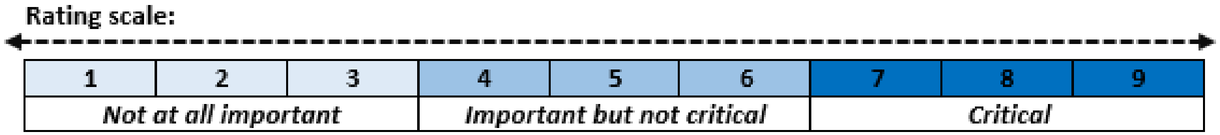

8. TREATMENT SATISFACTION

How the treatment meets your expectations or how pleased you are after receiving the treatment; or how likely you are to recommend the treatment

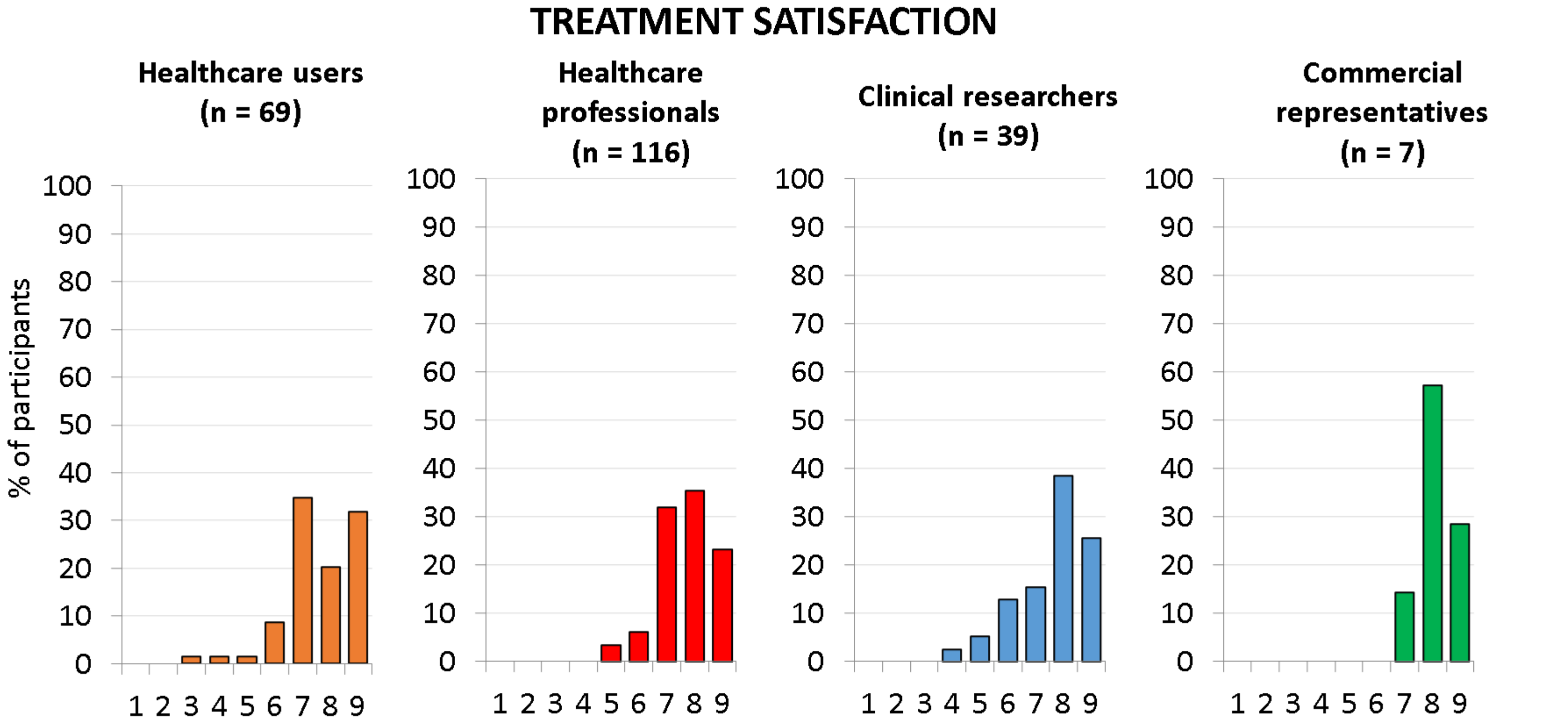

Domain Category: Factors related to the treatment being tested

Rating scale:

1	2	3	4	5	6	7	8	9
Not at all important			*Important but not critical*			*Critical*		

NIHR | National Institute for Health Research

9. DEVICE USAGE

How you use the device
(for example; in what situations; for how long)

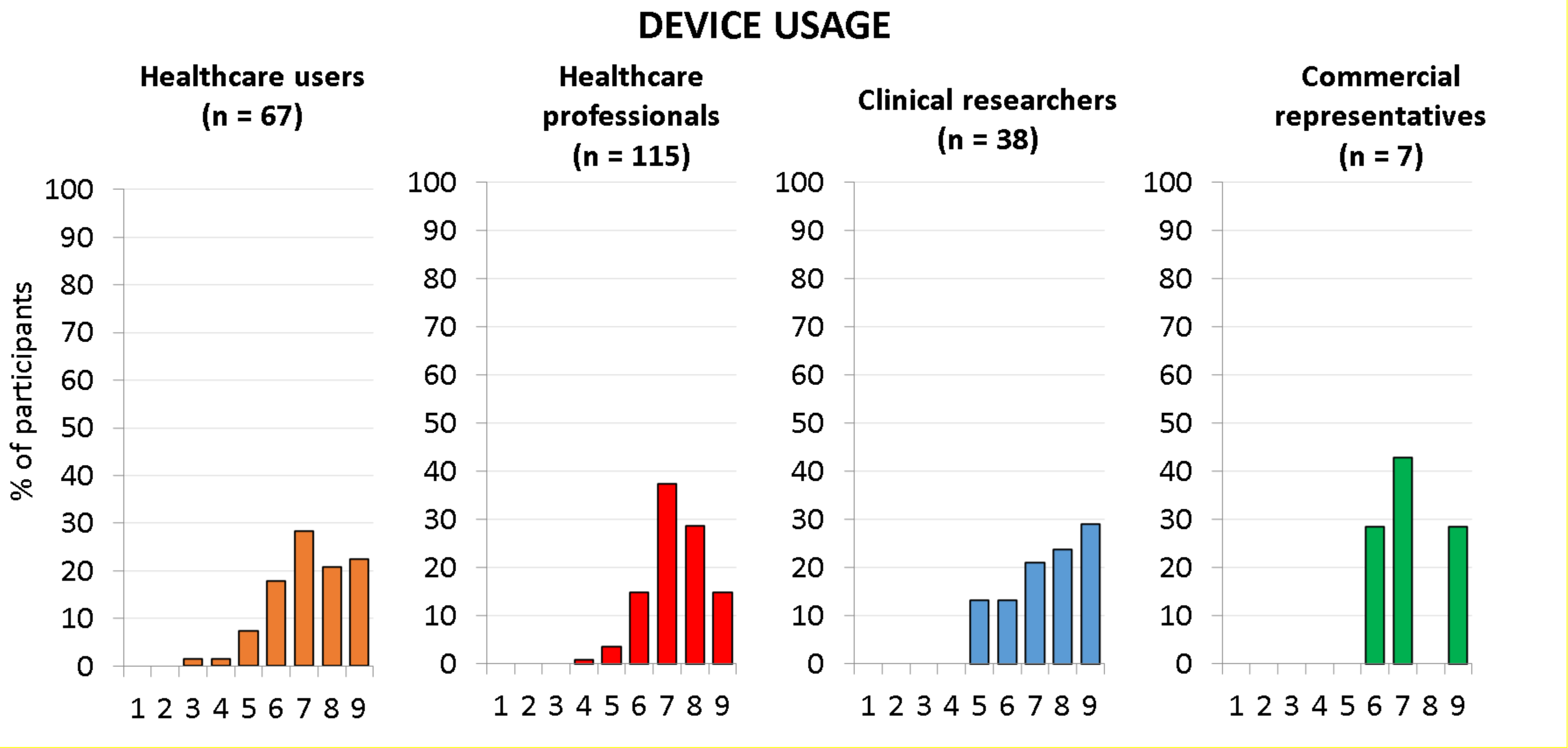

Domain Category: Factors related to the treatment being tested

NIHR | National Institute for Health Research

Rating scale:

1	2	3	4	5	6	7	8	9
Not at all important			*Important but not critical*			*Critical*		

10. DEVICE MALFUNCTION

The device does not work as it should or it stops working

CROSSSD
CORE REHABILITATION OUTCOME SET for SINGLE SIDED DEAFNESS

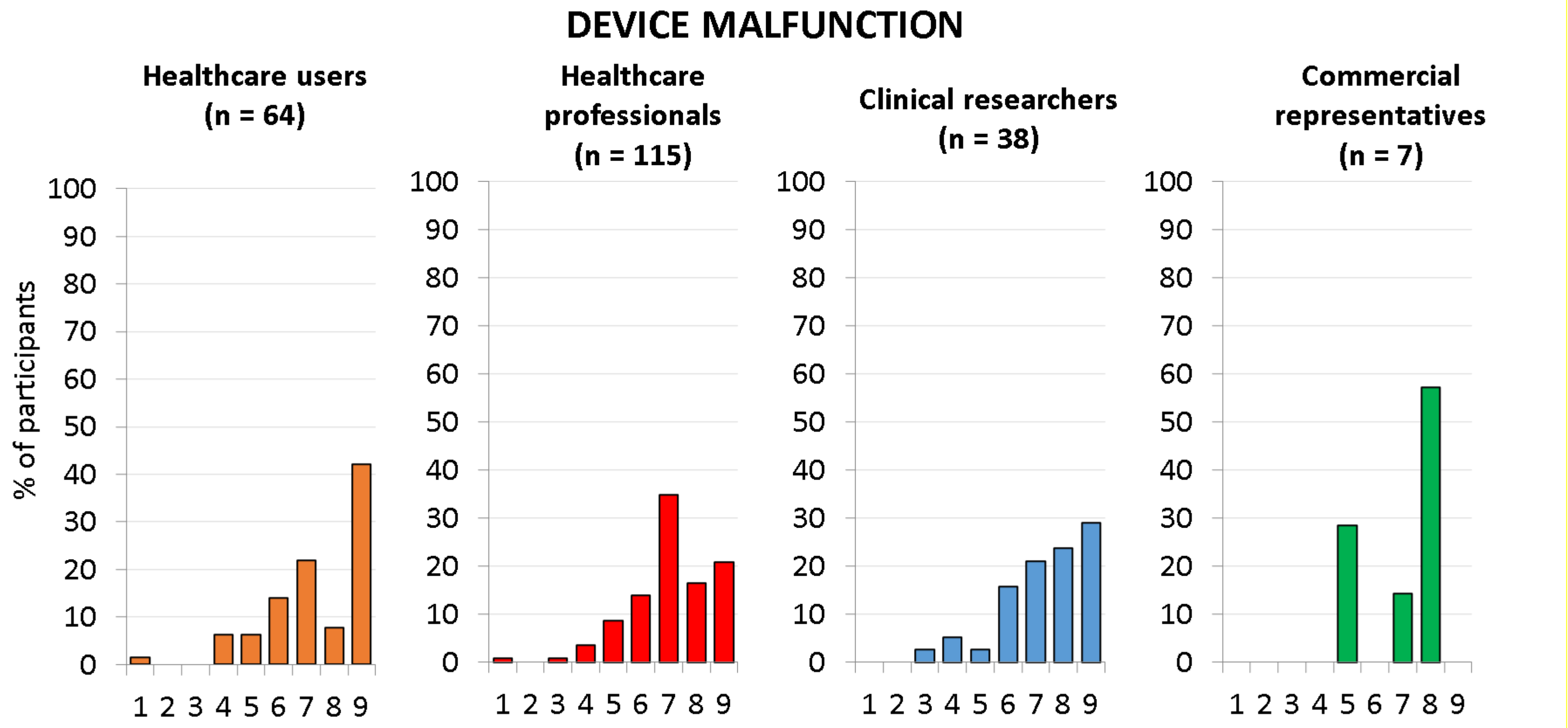

Domain Category: Factors related to the treatment being tested

NIHR | National Institute for Health Research

Rating scale:

1	2	3	4	5	6	7	8	9
Not at all important			*Important but not critical*			*Critical*		

12. AVOIDING SOCIAL SITUATIONS

Choosing not to go to particular social situations because of your hearing loss

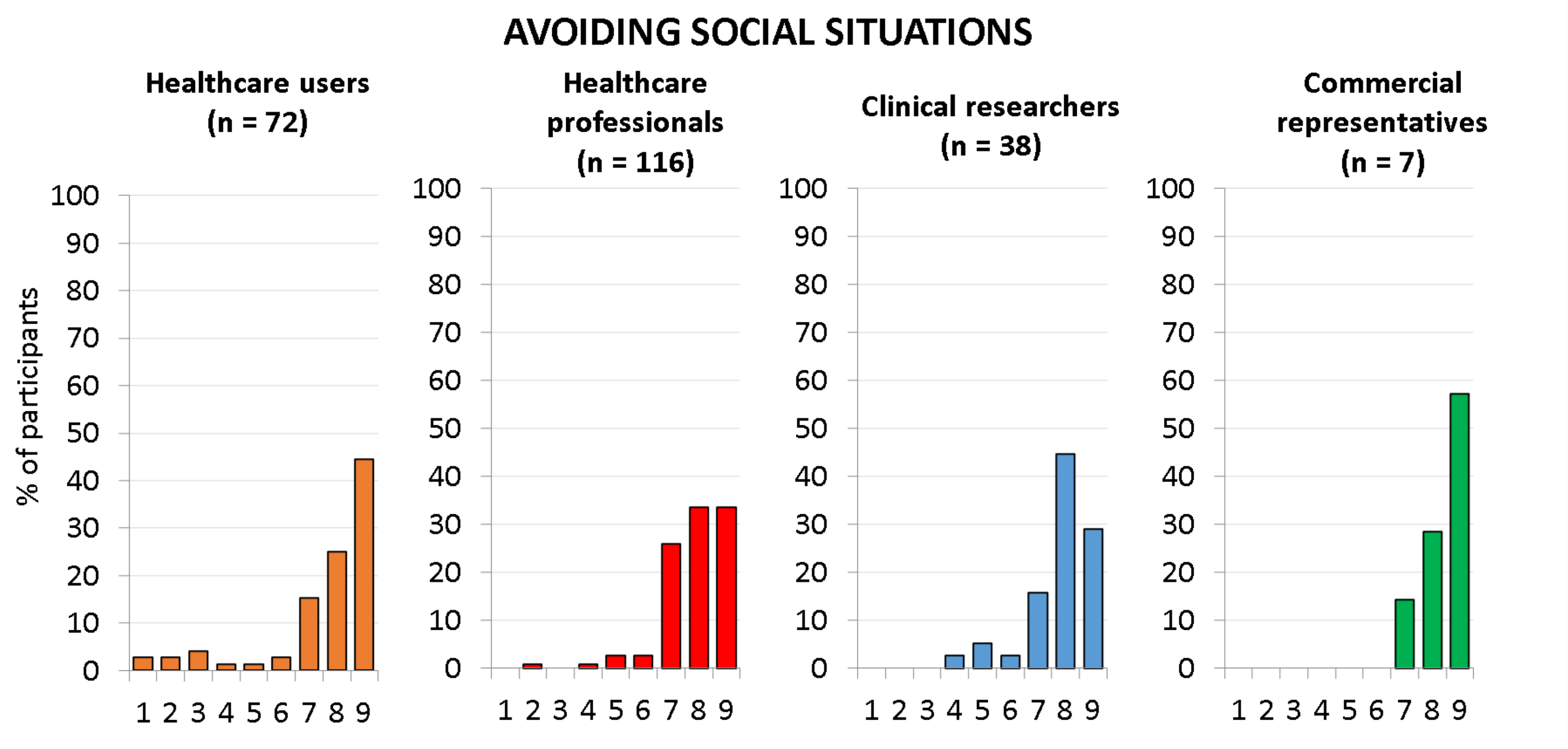

Domain Category:

Health-related quality of life

NIHR | National Institute for Health Research

Rating scale:

1	2	3	4	5	6	7	8	9
Not at all important			*Important but not critical*			*Critical*		

15. IMPACT ON SOCIAL SITUATIONS

Your hearing loss or device limiting your ability to fully participate in the social world; especially in challenging situations or where a lot of effort is needed to follow the conversation (for example; at a restaurant; at the park; in a bar or at a party)

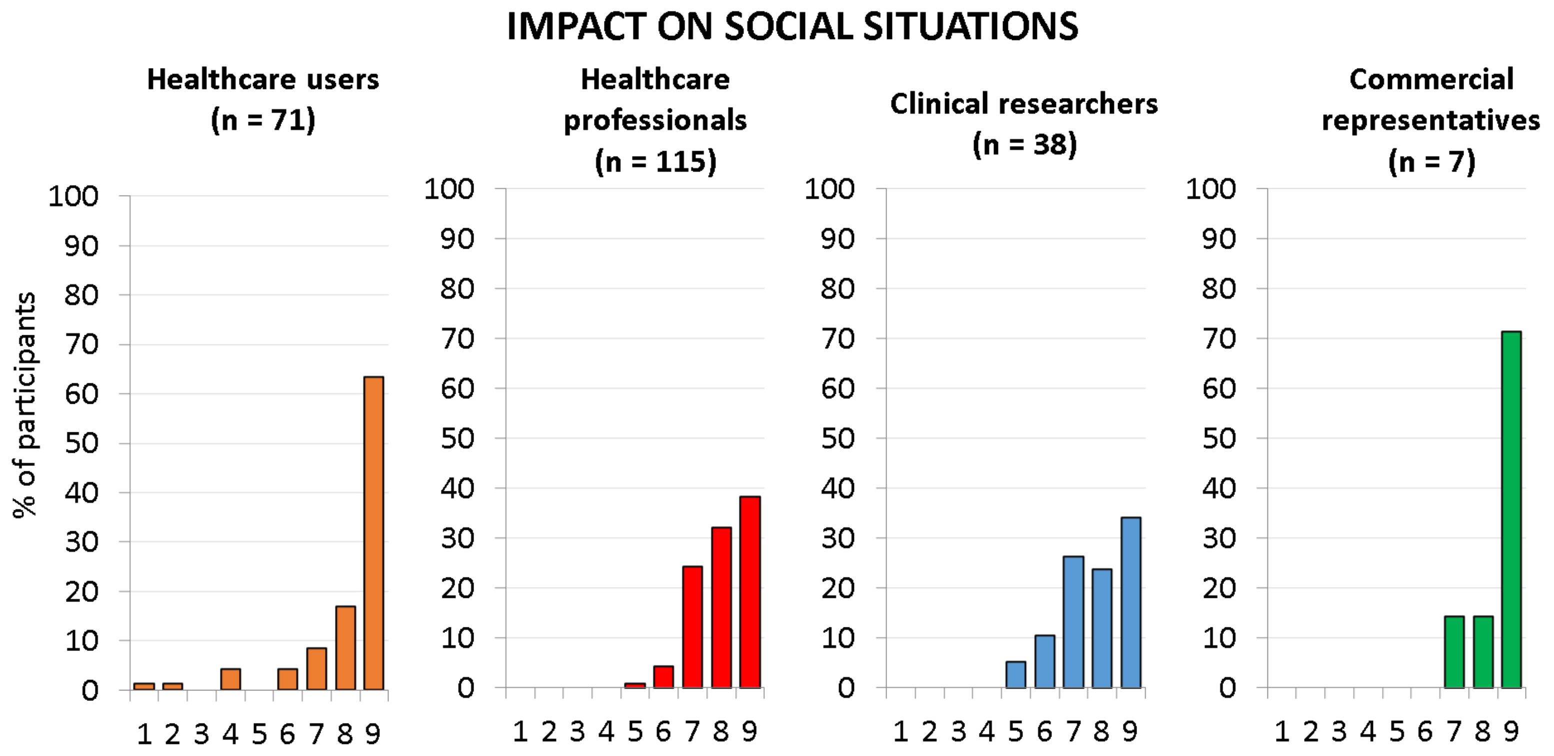

Domain Category:

Health-related quality of life

Rating scale:

1	2	3	4	5	6	7	8	9
Not at all important			*Important but not critical*			*Critical*		

NIHR | National Institute for Health Research

16. IMPACT ON WORK

Effect of your hearing loss or device on your ability to carry out work tasks or job roles; or advancing your career

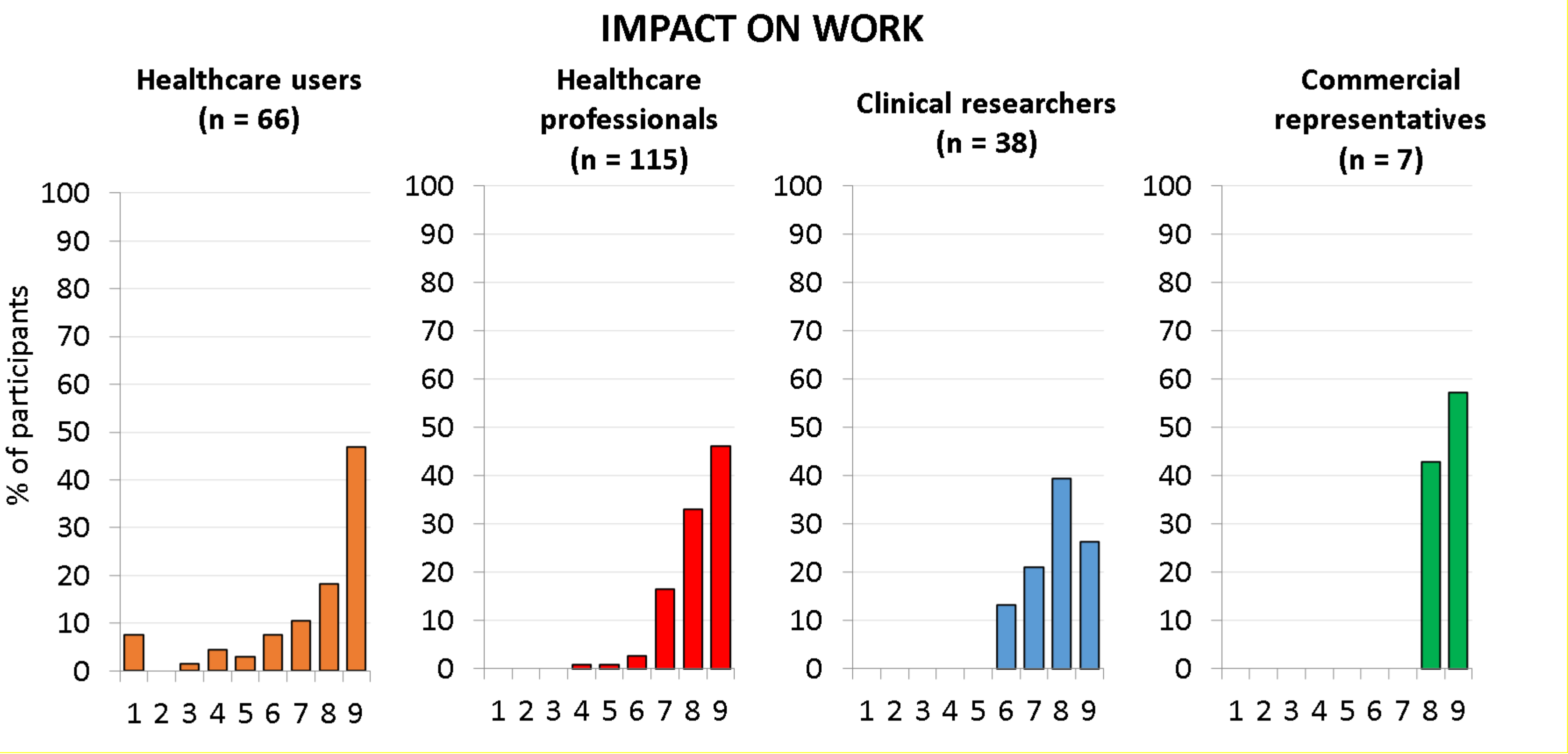

Domain Category:

Health-related quality of life

NIHR | National Institute for Health Research

Rating scale:

1	2	3	4	5	6	7	8	9
Not at all important			*Important but not critical*			*Critical*		

17. BEING AWARE OF A SOUND

Being aware of a sound and recognising what that sound is (for example; being aware that someone has started to speak)

CROSSSD
CORE REHABILITATION OUTCOME SET for SINGLE SIDED DEAFNESS

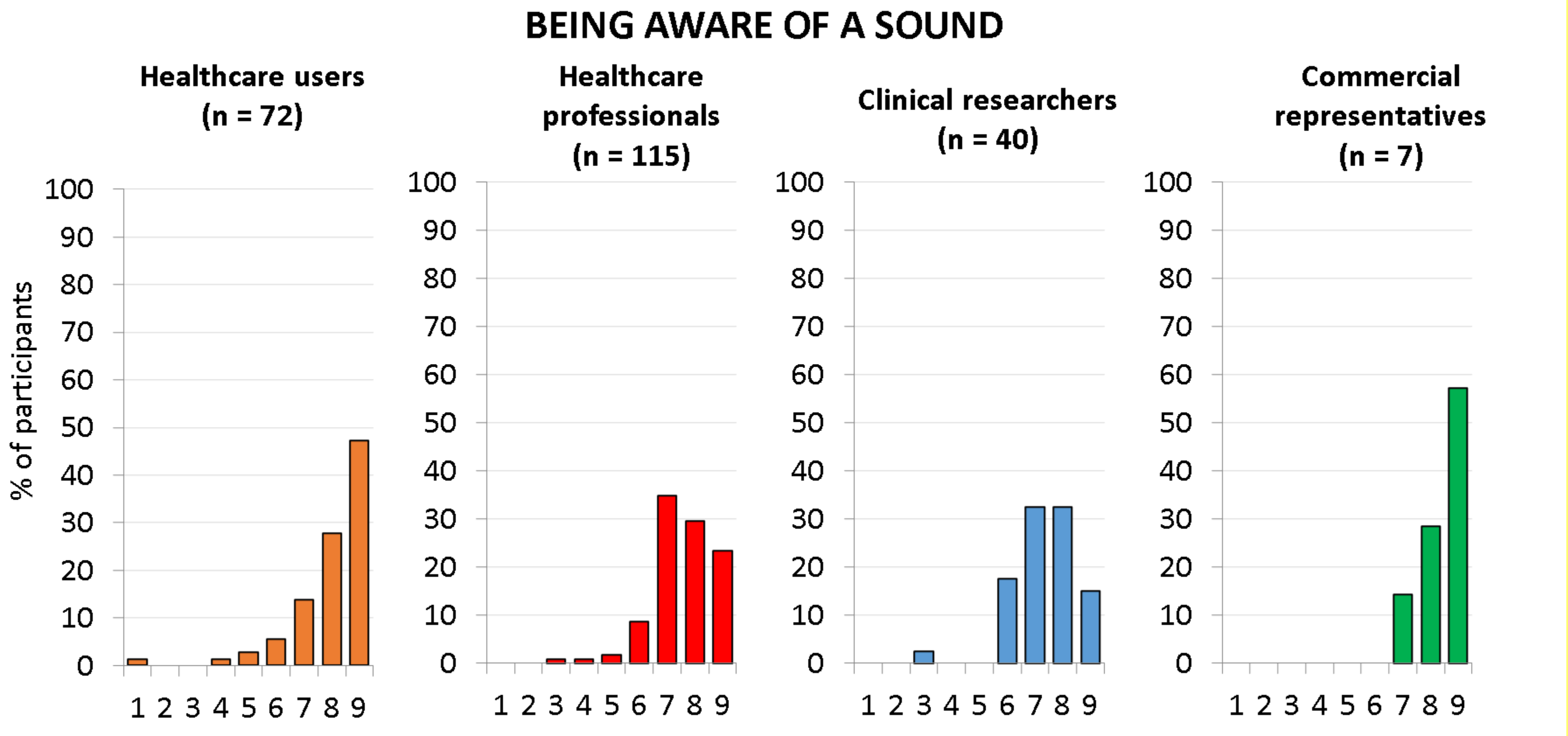

Domain Category:
Hearing disability

NIHR | National Institute for Health Research

Rating scale:

1	2	3	4	5	6	7	8	9
Not at all important			*Important but not critical*			*Critical*		

18. LISTENING IN COMPLEX SITUATIONS

The difficulty experienced when listening to a sound while separating it out from a background of other sounds

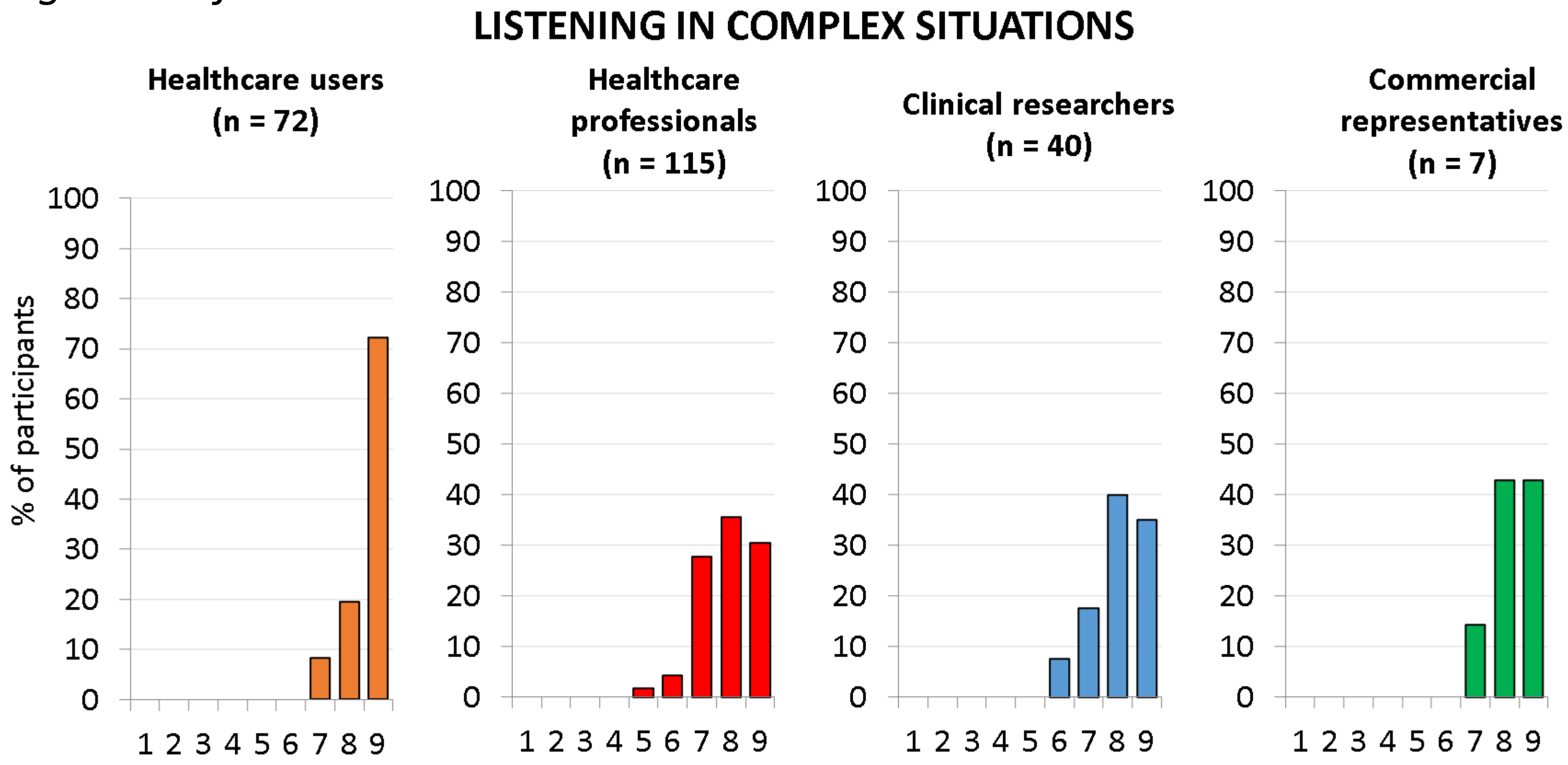

Domain Category:
Hearing disability

Rating scale:

1	2	3	4	5	6	7	8	9
Not at all important			*Important but not critical*			*Critical*		

NIHR | National Institute for Health Research

19. LISTENING IN REVERBERANT CONDITIONS

The difficulty experienced when listening in places where the sound reflects off the walls; floor or ceiling (echoes); creating a blurred sound. For example; understanding announcements in train stations or airports

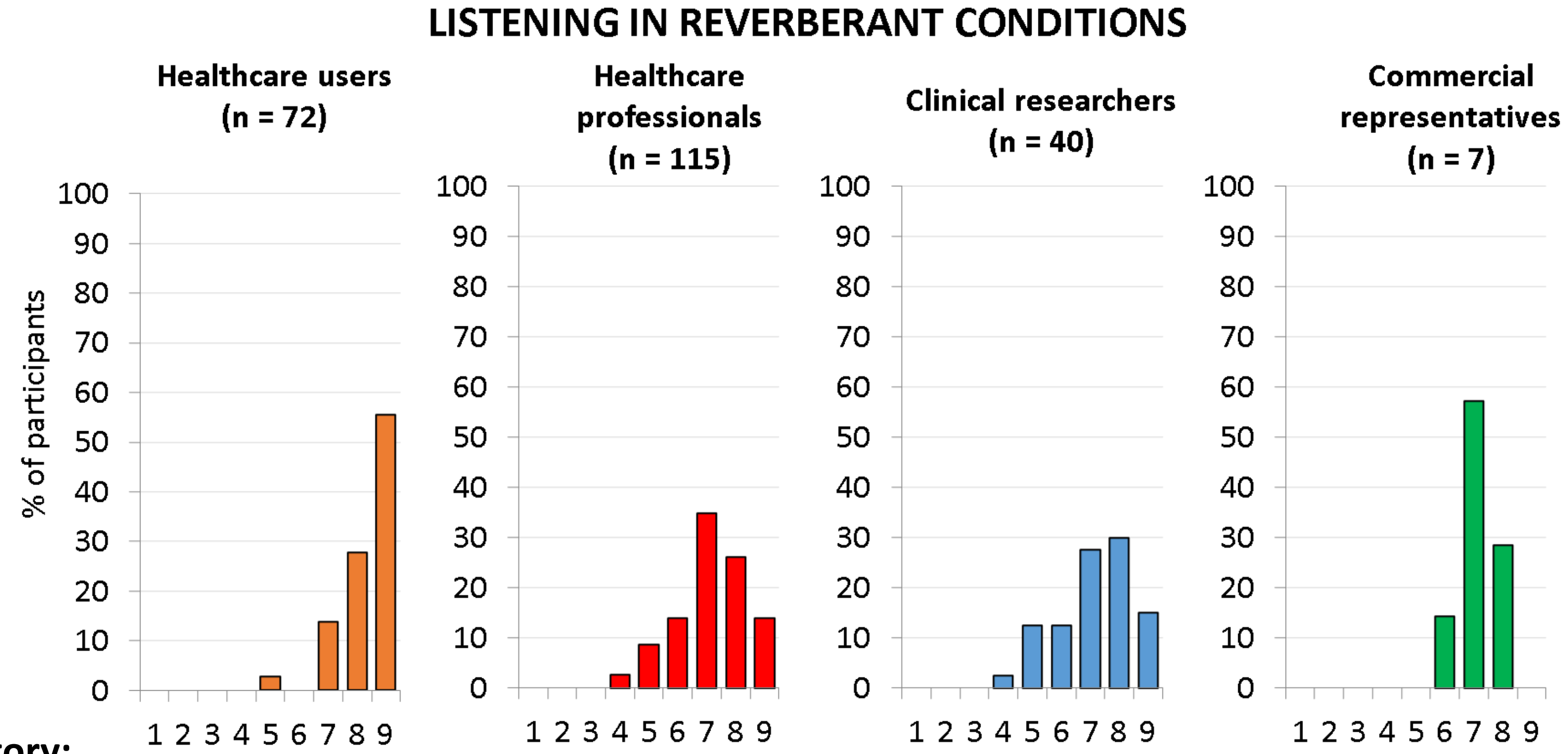

Domain Category:
Hearing disability

Rating scale:

1	2	3	4	5	6	7	8	9
Not at all important			*Important but not critical*			*Critical*		

NIHR | National Institute for Health Research

21. GROUP CONVERSATION IN QUIET

Listening and following a conversation between a group of people; in a quiet environment

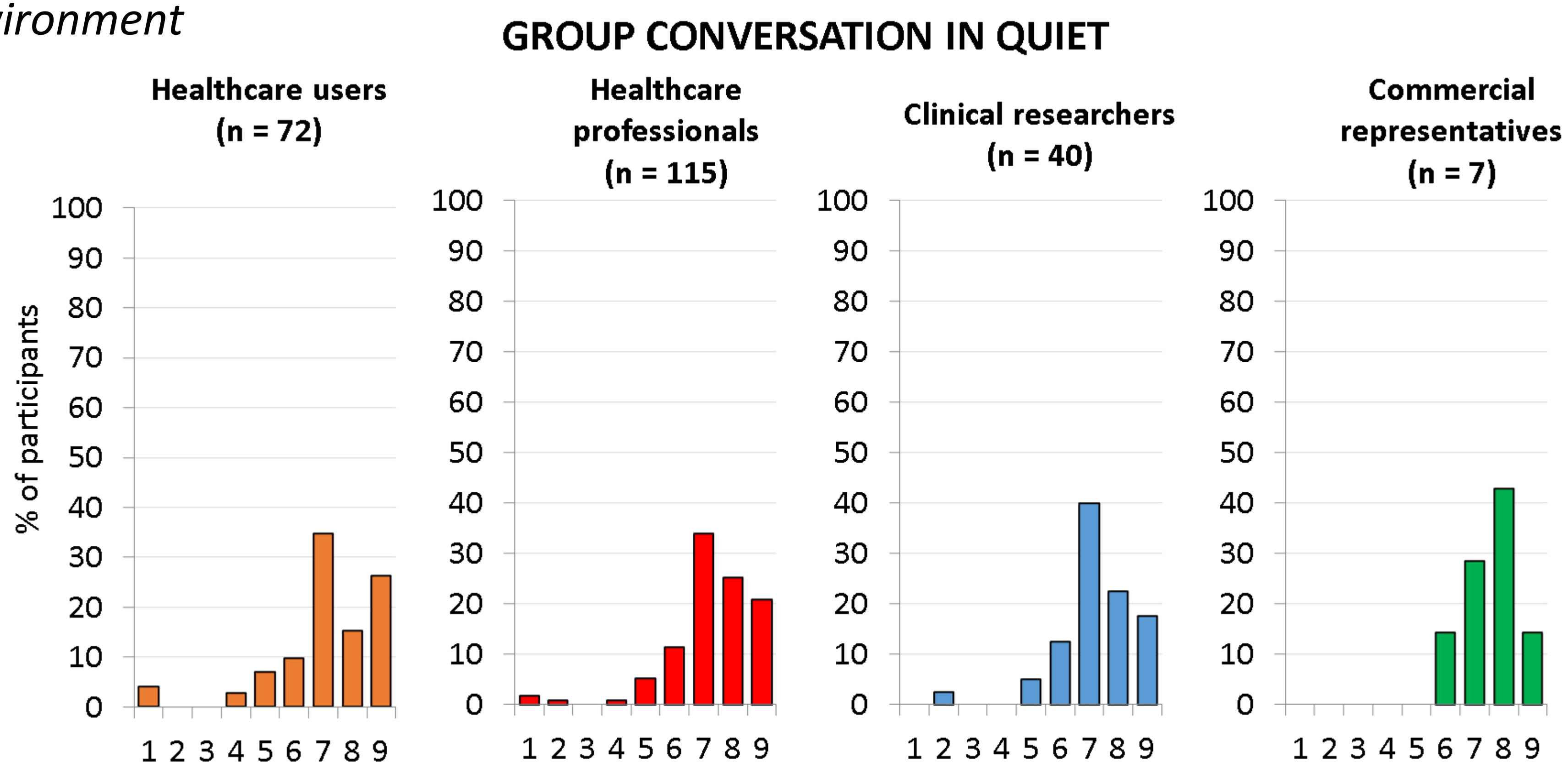

Domain Category:
Hearing disability

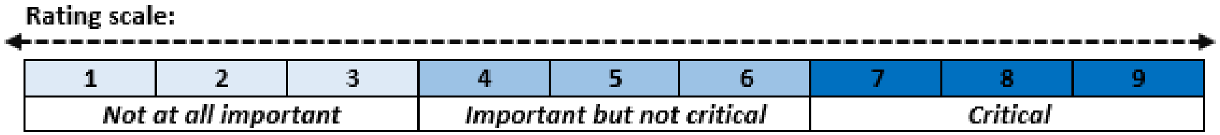

NIHR | National Institute for Health Research

22. ONE-TO-ONE CONVERSATION IN GENERAL NOISE

Listening and understanding one person; in a noisy environment

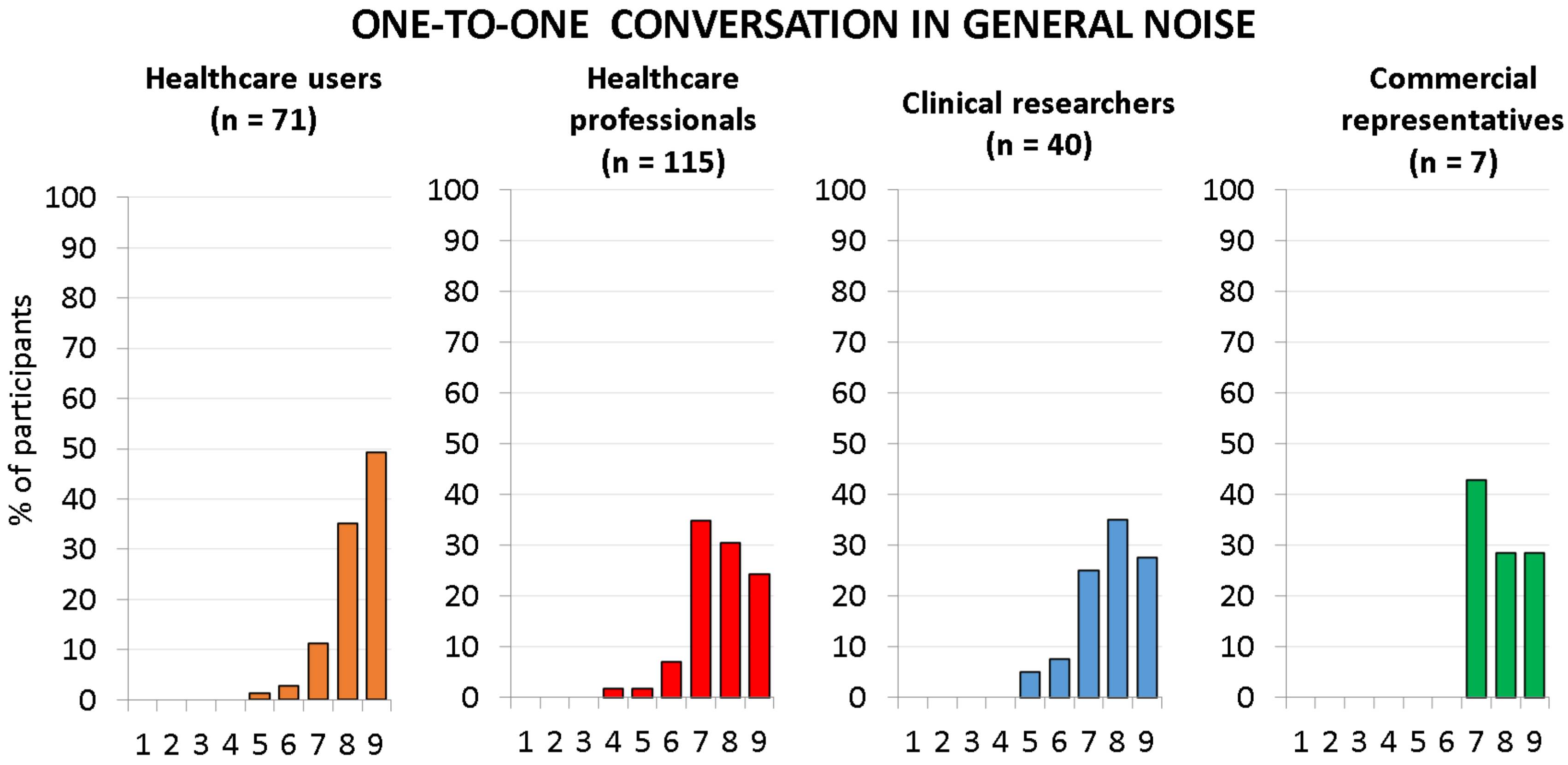

Domain Category:

Hearing disability

NIHR | National Institute for Health Research

Rating scale:

1	2	3	4	5	6	7	8	9
Not at all important			*Important but not critical*			*Critical*		

23. GROUP CONVERSATION IN NOISY SOCIAL SITUATIONS

Listening and following a conversation between a group of people; when others are talking in the background

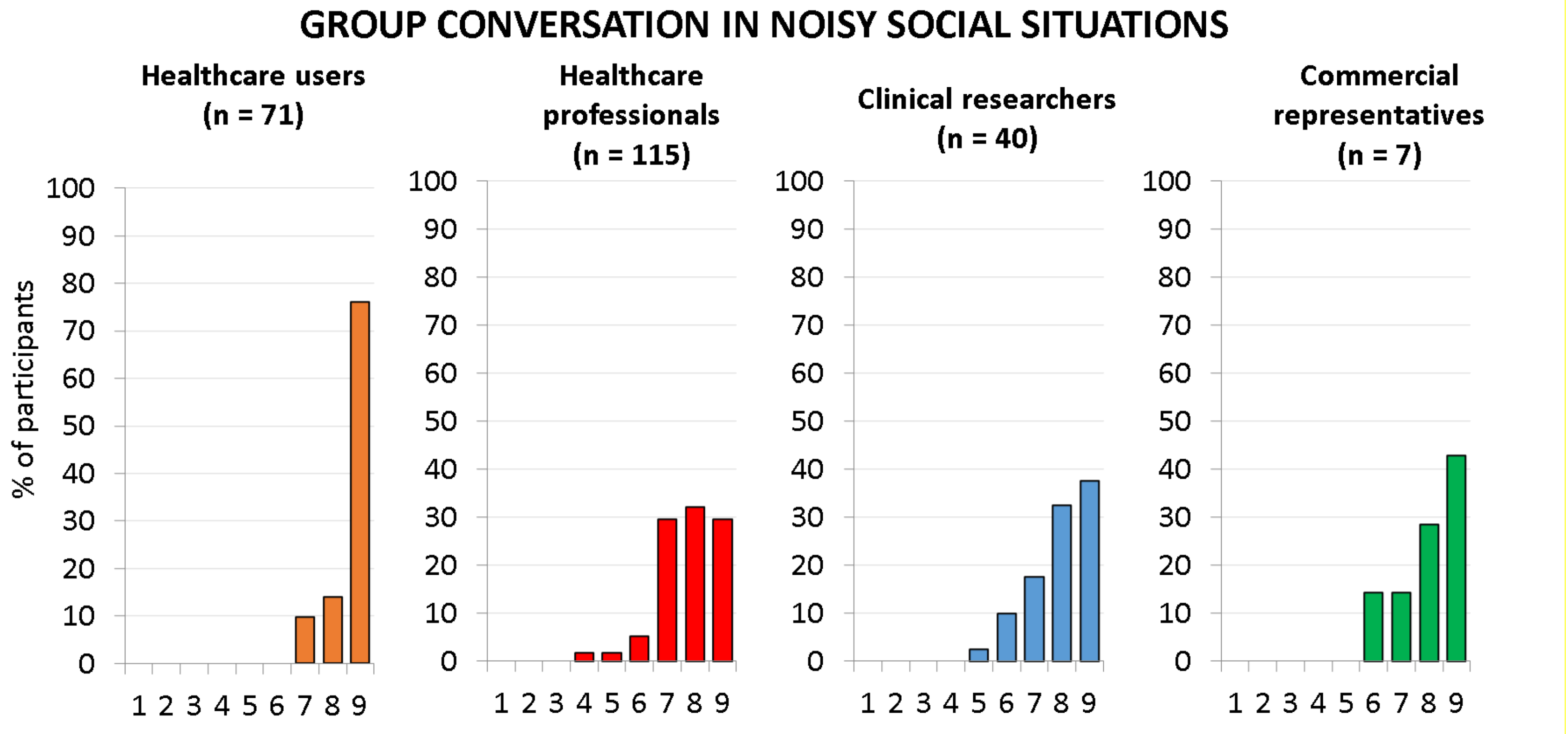

Domain Category:
Hearing disability

NIHR | National Institute for Health Research

Rating scale:

1	2	3	4	5	6	7	8	9
Not at all important			*Important but not critical*			*Critical*		

24. SOUND LOCALISATION

Knowing where a sound is coming from

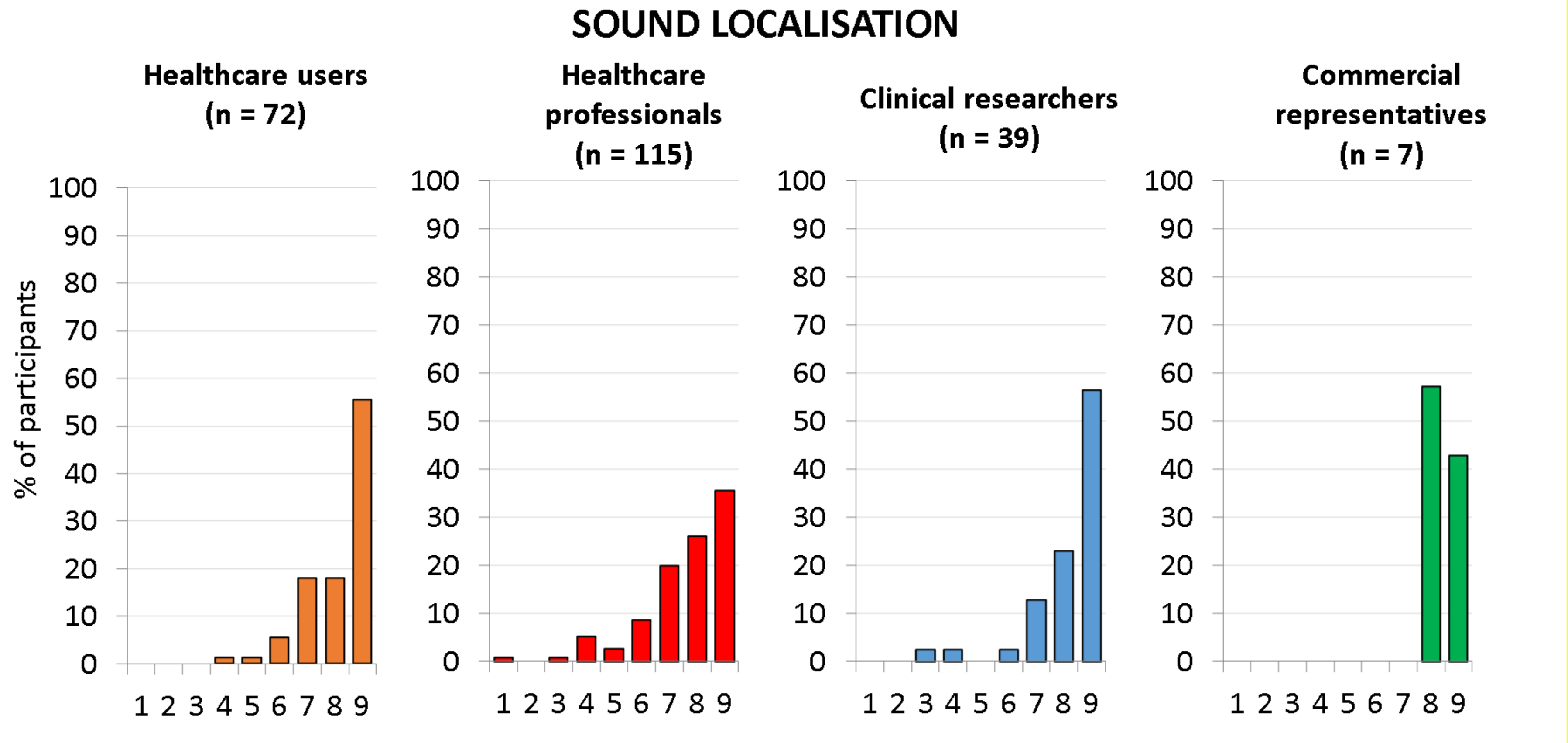

Domain Category:

Spatial hearing

NIHR | National Institute for Health Research

Rating scale:

1	2	3	4	5	6	7	8	9
Not at all important			*Important but not critical*			*Critical*		

26. SPATIAL ORIENTATION

Knowing where you are in relation to the position of a sound source

CROSSSD
CORE REHABILITATION OUTCOME SET for SINGLE SIDED DEAFNESS

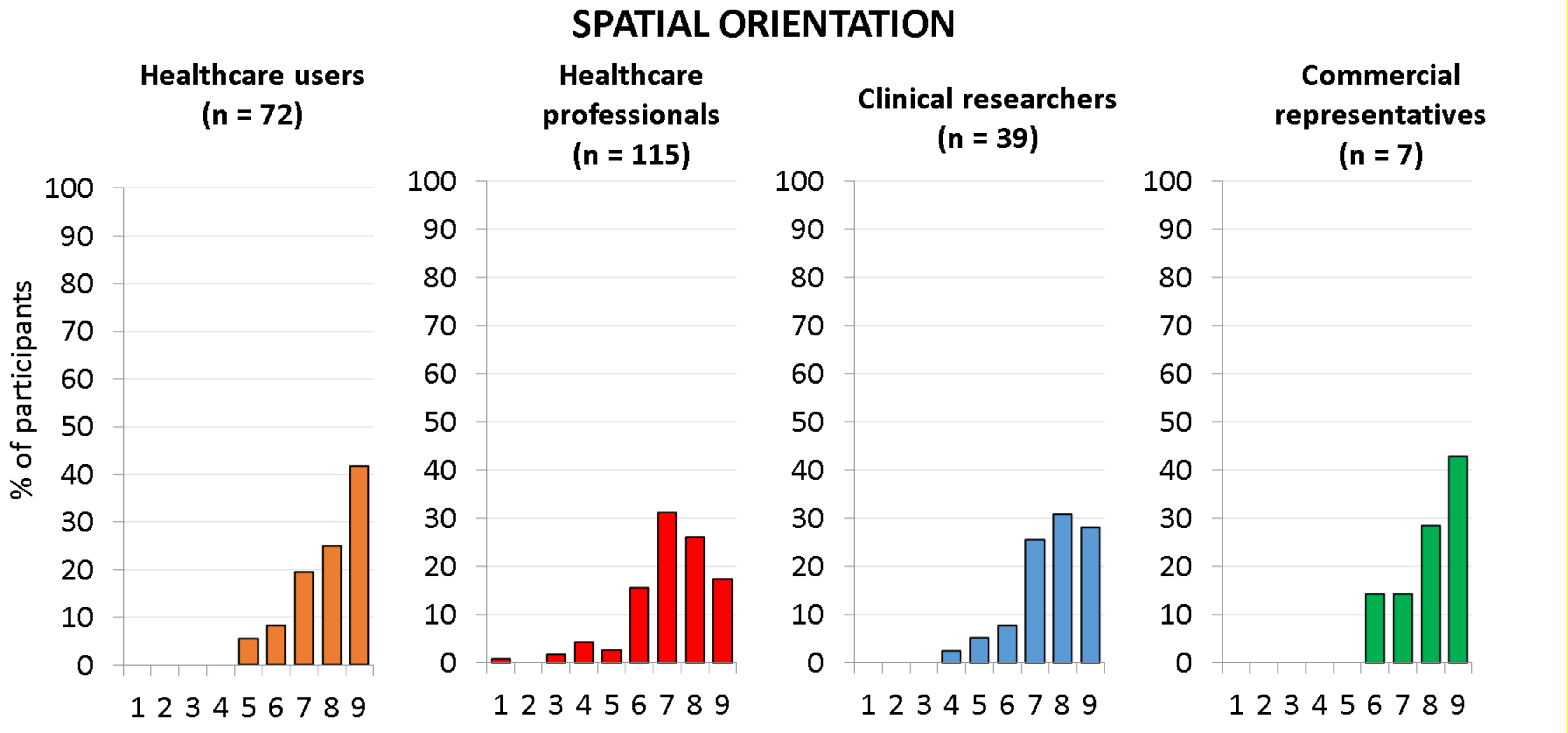

Domain Category:

Spatial hearing

NIHR | National Institute for Health Research

Rating scale:

1	2	3	4	5	6	7	8	9
Not at all important			*Important but not critical*			*Critical*		

28. PHYSICAL TIREDNESS

Tiredness or fatigue from the effort of listening or when you need to turn your head repeatedly to listen in social situations

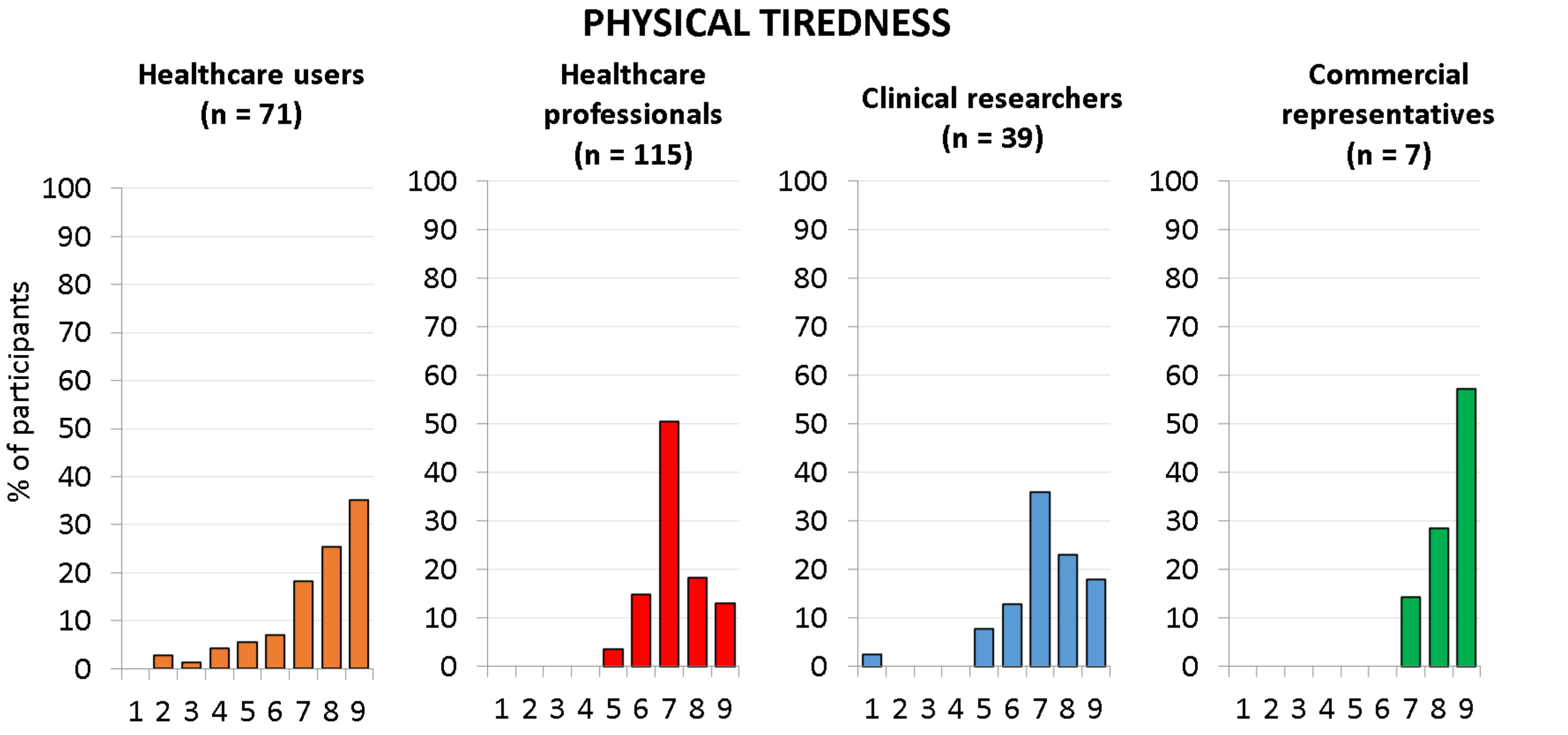

Domain Category:

Physical effects

Rating scale:

1	2	3	4	5	6	7	8	9
Not at all important			*Important but not critical*			*Critical*		

NIHR National Institute for Health Research

35. PERSONAL SAFETY

How your hearing loss effects your awareness of potential hazards and threats in your daily life (for example; moving traffic; hazards at the workplace) and those you may not be able to see or hear (for example; other people behind you)

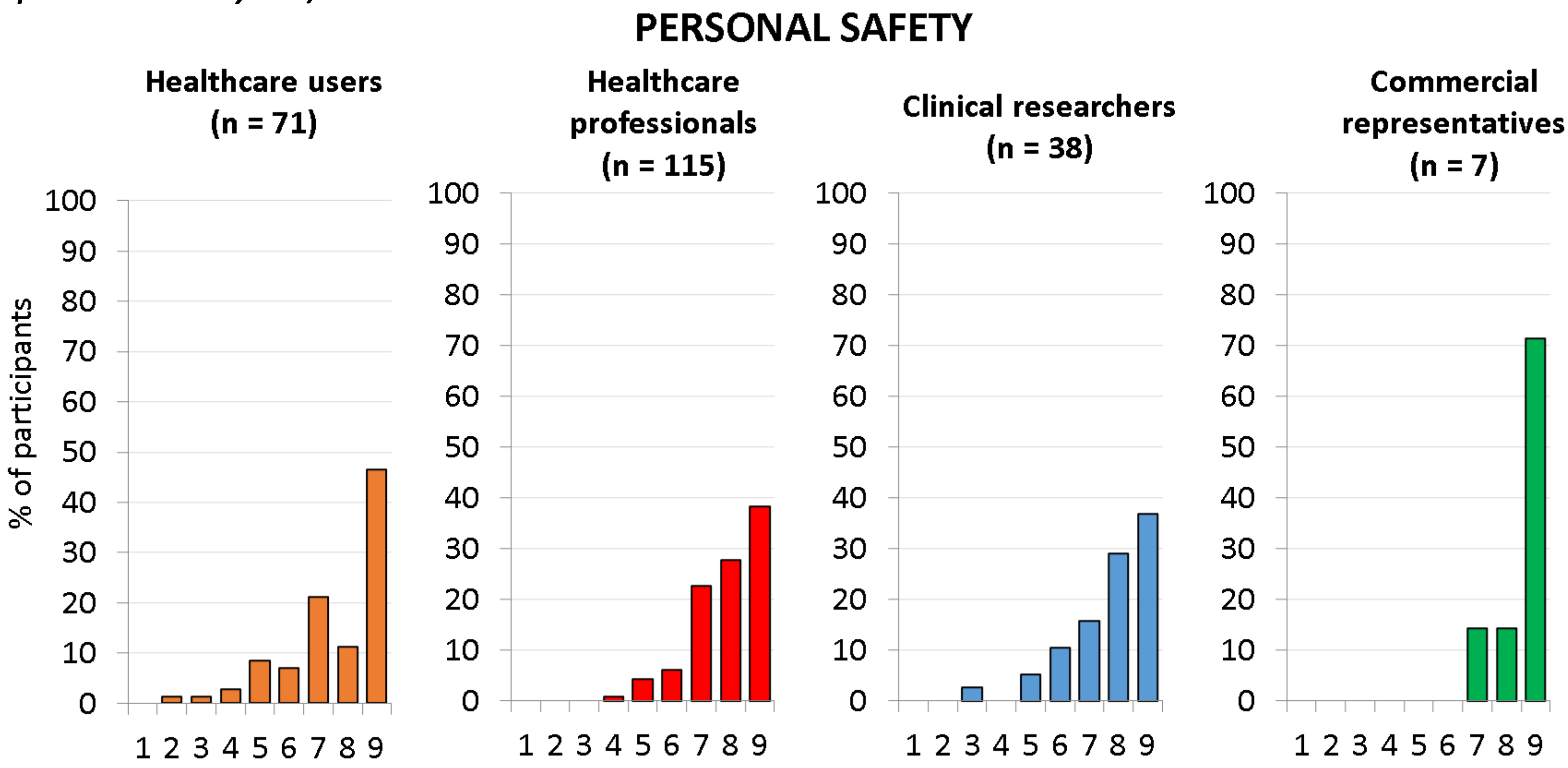

Domain Category: Self

NIHR | National Institute for Health Research

Rating scale:

1	2	3	4	5	6	7	8	9
Not at all important			*Important but not critical*			*Critical*		

CROSSSD
CORE REHABILITATION OUTCOME SET for SINGLE SIDED DEAFNESS

List of the 49 outcome domains that were rated during the second round of the Delphi survey for Inclusion, Exclusion or 'Maybe'

e-Delphi survey results

CROSSSD

Round 1 e-Delphi (9th Sept - 21st Nov):

n=44 outcome domains to rate
n=308 registrations
n=272 rated all domains
n=36 did not complete
n=95 suggested additional outcomes
n=5 outcomes added to Round 2

Round 2 e-Delphi (21st Nov - 24th Feb):

n=49 outcome domains to rate
n=233 rated all domains
n=44 did not complete
n=870 changes to Round 1 ratings

Stakeholder group participants

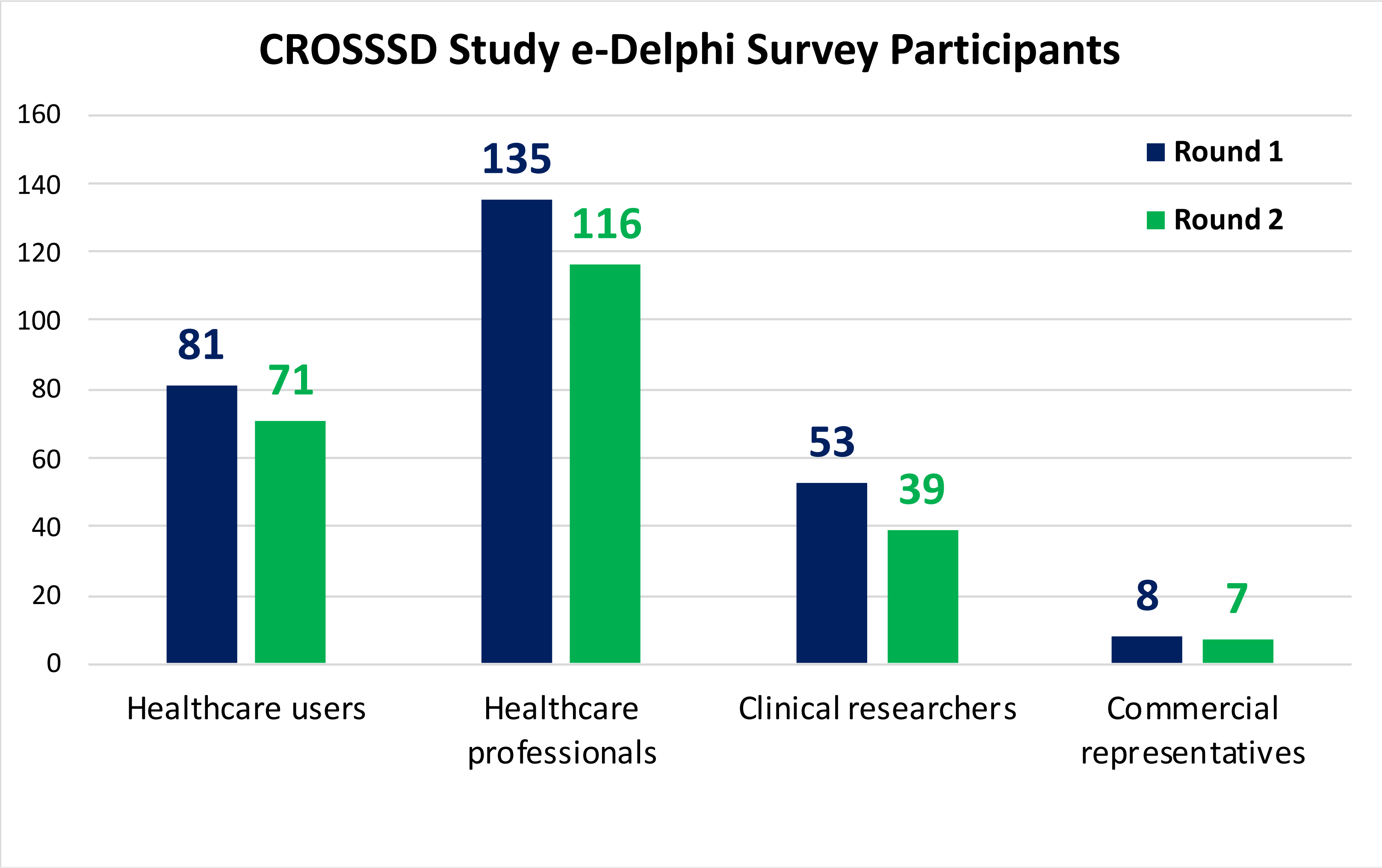

Rules: Outcome Inclusion

Outcomes where at least 70% scored 7-9 and less than 15% scored 1-3 in all stakeholder groups

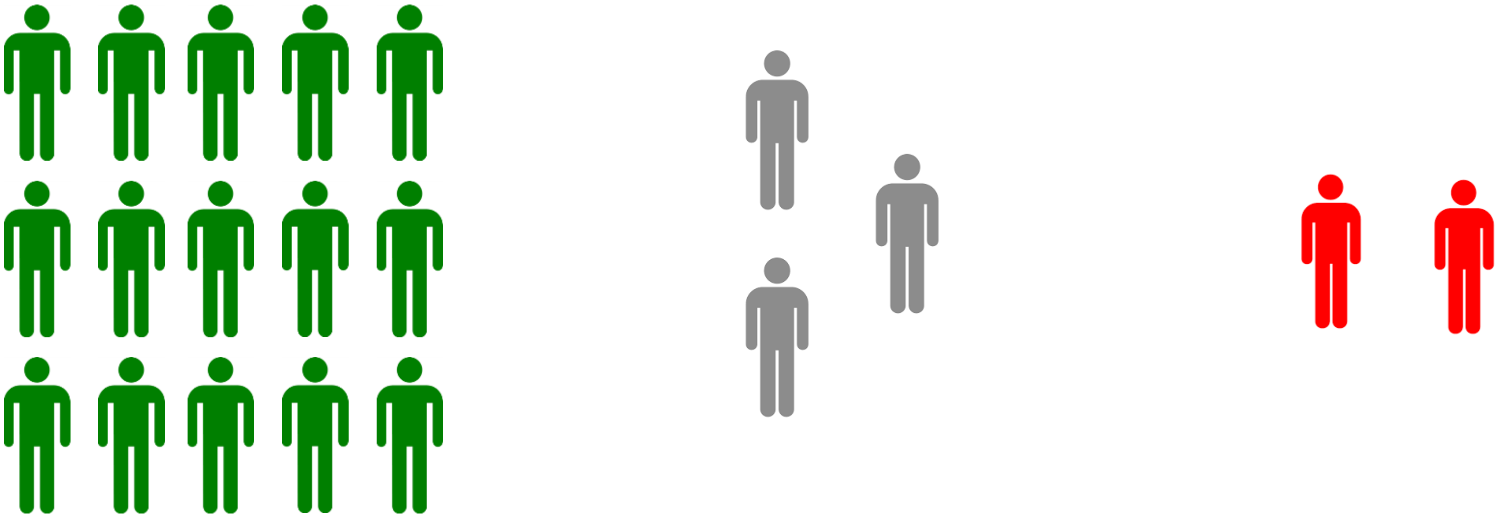

at least 70% scored it as ‘critically important’

less than 15% scored it ‘unimportant’

Outcomes for Inclusion

CROSSSD
CORE REHABILITATION OUTCOME SET for SINGLE SIDED DEAFNESS

Domain category	Outcome	Outcome definition
Other effects	**7. Listening effort**	Exerting greater effort to listen and follow a conversation. This might consequently lead to feelings of tiredness and fatigue; but those feelings would be a separate outcome domain
Factors related to the treatment being tested	**8. Treatment satisfaction**	How the treatment meets your expectations or how pleased you are after receiving the treatment; or how likely you are to recommend the treatment
	**9. Device usage**	How you use the device (for example; in what situations; for how long)
	**10. Device malfunction**	The device does not work as it should or it stops working
Health-related quality of life	**12. Avoiding social situations**	Choosing not to go to particular social situations because of your hearing loss
	**15. Impact on social situations**	Your hearing loss or device limiting your ability to fully participate in the social world; especially in challenging situations or where a lot of effort is needed to follow the conversation (for example; at a restaurant; at the park; in a bar or at a party)
	**16. Impact on work**	Effect of your hearing loss or device on your ability to carry out work tasks or job roles; or advancing your career

Outcomes for Inclusion

Domain category	Outcome	Outcome definition
Hearing disability	**17. Being aware of a sound**	Being aware of a sound and recognising what that sound is (for example; being aware that someone has started to speak)
	**18. Listening in complex situations**	The difficulty experienced when listening to a sound while separating it out from a background of other sounds
	**19. Listening in reverberant conditions**	The difficulty experienced when listening in places where the sound reflects off the walls; floor or ceiling (echoes); creating a blurred sound. For example; understanding announcements in train stations or airports
	**21. Group conversation in quiet**	Listening and following a conversation between a group of people; in a quiet environment
	**22. One-to-one conversation in general noise**	Listening and understanding one person; in a noisy environment
	**23. Group conversation in noisy social situations**	Listening and following a conversation between a group of people; when others are talking in the background
Spatial hearing	**24. Sound localisation**	Knowing where a sound is coming from
	**26. Spatial orientation**	Knowing where you are in relation to the position of a sound source
Physical effects	**28. Physical tiredness**	Tiredness or fatigue from the effort of listening or when you need to turn your head repeatedly to listen in social situations
Self	**35. Personal safety**	How your hearing loss effects your awareness of potential hazards and threats in your daily life (for example; moving traffic; hazards at the workplace) and those you may not be able to see or hear (for example; other people behind you)

Rules: Outcome Exclusion

CROSSSD

Outcomes where less than 50% of participants in all stakeholder groups scored 7-9

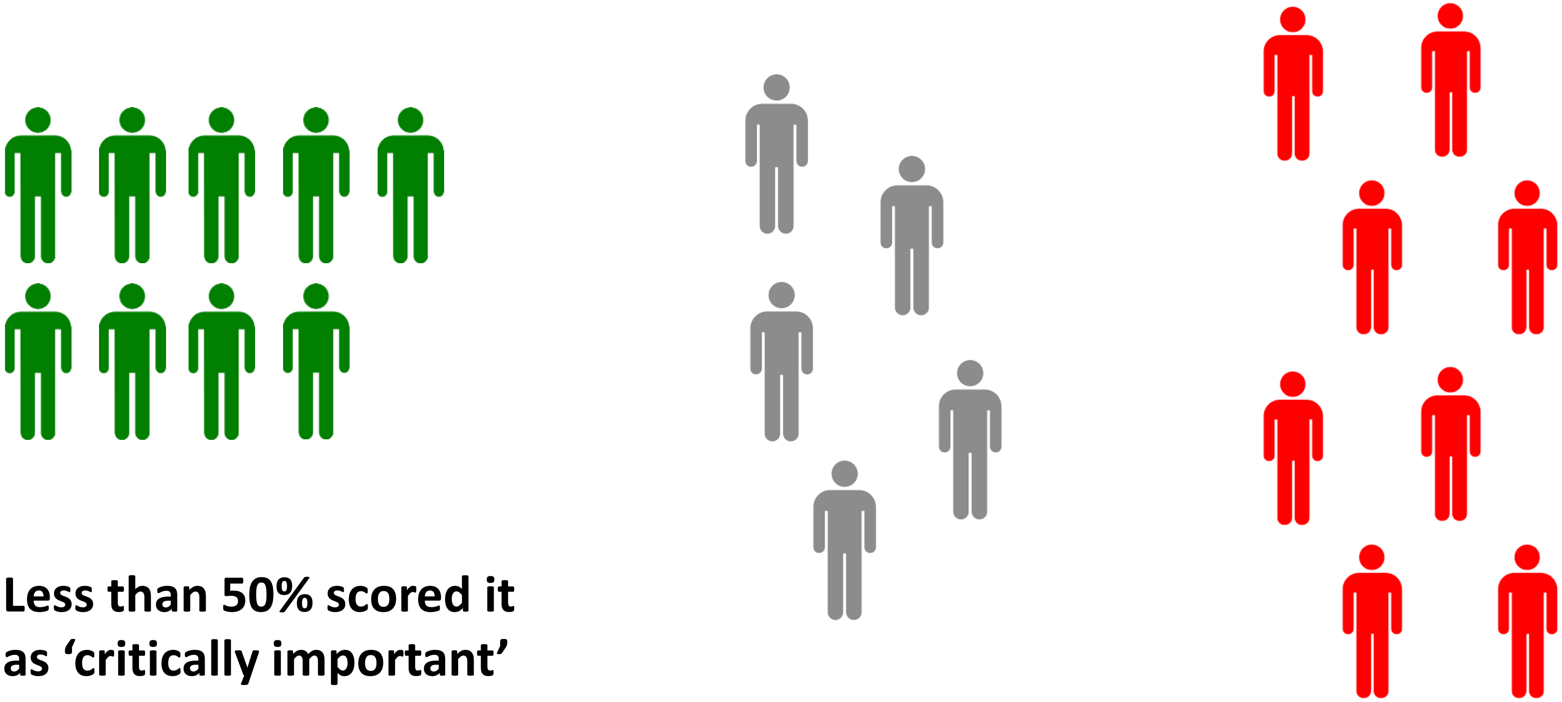

Less than 50% scored it as ‘critically important’

Outcomes for Exclusion

Domain category	Outcome	Outcome definition
Psychological effects	**2. Discomfort in listening situations**	Finding yourself in listening situations that you feel you can't adequately control (for example; when you can't choose a favourable listening position); or situations in which you don't feel comfortable (for example when interacting with people who don't know you have a hearing loss)
	**3. Emotional distress**	A negative unpleasant emotional reaction which may include fear; anger; frustration; anxiety; and suffering
	**4. Mood**	General sense of well-being; ranging from feeling very low or negative to very positive
	**5. Motivation**	A willingness to engage in challenging listening situations
Factors related to the treatment being tested	**11. Adverse events**	Any bad or unexpected thing that happens during the time a treatment is being tested in a clinical trial
Health-related quality of life	**13. Impact on individual activities**	Effect of your hearing loss or your device on your choice to engage in individual activities (for example; travelling alone; swimming or watching TV / films / movies)
	**14. Impact on relationships**	Effect of your hearing loss or your device on making new relationships and maintaining relationships with a spouse or partner; family; friends and colleagues

Outcomes for Exclusion

CROSSSD
CORE REHABILITATION OUTCOME SET for SINGLE SIDED DEAFNESS

Domain category	Outcome	Outcome definition
Spatial hearing	**25. Sound distance**	Knowing if a sound is close by or far away
Self	**35. Self-Image**	Feeling incomplete or incapable because you are unable to do all the things that you want to do
Sound quality	**37. Loudness**	How 'loud' a sound seems to you
	**38. Fullness**	How 'full' a sound seems to you. This can also be described as the 'richness'; 'warmth' or 'depth' of a sound
	**39. Clarity**	How 'clear' a sound seems to you
Tinnitus	**41. Tinnitus intrusiveness**	Being acutely aware of the sounds of tinnitus; feeling that it is invading your life or your personal space; changing your thoughts or actions and negatively impacting on your life
	**42. Tinnitus loudness**	How loud your tinnitus sounds

Outcomes for Exclusion

Domain category	Outcome	Outcome definition
Factors related to the treatment being tested	**45. Device usability**	How easy it is to learn; use; and maintain the device (for example; changing the batteries; cleaning)
Health-related quality of life	**46. Impact on learning**	Effect of your hearing loss or device on your ability to acquire new knowledge or skills; or further your education
Psychological effects	**47. Independence**	How your hearing loss affects how much you need to rely on other people in daily life
Self	**48. Concern about your hearing**	Feeling worried about the hearing in your better ear and the thought that it may decline
	**49. Valnerability**	Feeling insecure because your hearing loss affects your awareness of potential hazards and threats in your daily life (for example; moving traffic; hazards at the workplace) and those you may not be able to see or hear (for example; other people behind you)

Maybes? No consensus

Domain category	Outcome	Outcome definition
Psychological effects	**6. Dissatisfaction with life**	Being unhappy because you feel you should be achieving or should have achieved more in your life
Physical effects	**30. Manual dexterity**	Having the fine motor skills needed to use your device effectively (for example; putting the device on; changing the batteries)
	**31. Tinnitus-related brain changes**	Changes in brain structure or function associated with tinnitus
	**32. Hearing-related brain changes**	Changes in brain structure or function associated with hearing loss
Self	**33. Self-stigma**	Negative perception of yourself due to your hearing loss and feeling stigmatised for using a hearing aid
Tinnitus	**40. Tinnitus awareness**	Noticing the sound of tinnitus is there
	**43. Tinnitus pitch**	Whether your tinnitus has a note-like quality; for example high pitch like whistling or low pitch like humming
	**44. Tinnitus quality**	What type of sound is heard (for example; hissing; buzzing; ringing; whistling etc)
