# Supplementary material for: Redesigning a Web-Based Stakeholder Consensus Meeting About Core Outcomes for Clinical Trials: Formative Feedback Study
Source: JMIR Form Res. 2021 Aug 19;5(8):e28878. doi: 10.2196/28878 (PMC8414289; doi:10.2196/28878)
Supplement: Multimedia Appendix 5 [file formative_v5i8e28878_app5.pdf]

(Form to be printed on local headed paper)

EVALUATION FORM

(Final Version 1.0: 22/05/2019)

IRAS Project ID: 239750

Title of Study: Core Rehabilitation Outcome Set in Single Sided Deafness (CROSSSD) Study

Name of Researcher: Roulla Katiri

Thank you for attending the CROSSSD study consensus meeting.

We would value your feedback about the consensus meeting, to help improve future core outcome set work. If you could take a few moments to let us know your thoughts, it would be much appreciated.

If you have any questions or would like more information, please contact Roulla Katiri (details below).

We would like to thank you for taking the time to complete this evaluation form.

Roulla Katiri, PhD Student
Tel: +44 (0) 115 823 2600
Email: roulla.katiri@nottingham.ac.uk
Web: www.nottingham.ac.uk/go/CROSSSD
National Institute for Health Research
Nottingham Hearing Biomedical Research Centre
@CROSSSD_
#CROSSSD

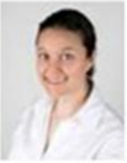

1. Please choose the option which describes you best:

☐ Health care professional ☐ Patient

2. The information that the organisers provided me with in advance of the meeting was helpful.

☐ Strongly agree ☐ Agree ☐ Neither ☐ Disagree ☐ Strongly disagree

Comments:

3. I was satisfied with the process used to agree the core outcomes set on the meeting day.

☐ Strongly agree ☐ Agree ☐ Neither ☐ Disagree ☐ Strongly disagree

Comments:

4. I was satisfied with the way the meeting was facilitated.
☐ Strongly agree ☐ Agree ☐ Neither ☐ Disagree ☐ Strongly disagree
Comments:

5. I felt able to contribute to the meeting.
☐ Strongly agree ☐ Agree ☐ Neither ☐ Disagree ☐ Strongly disagree
Comments:

6. I felt comfortable in communicating my views.
☐ Strongly agree ☐ Agree ☐ Neither ☐ Disagree ☐ Strongly disagree
Comments:

7. The workshop produced a fair result.
☐ Strongly agree ☐ Agree ☐ Neither ☐ Disagree ☐ Strongly disagree
Comments:

8. Do you have any comments about the practical arrangements for the workshop (e.g. venue, timing of the meeting, catering, number of breaks, or anything else)?

9. Was there anything else that could have been done to improve the workshop?
